# Multi-scale analyses on performance degradation of reinforced concrete structure due to damage evolution on bonding interface

**DOI:** 10.1371/journal.pone.0214915

**Published:** 2019-04-16

**Authors:** Ying Wang, Yuqian Zheng, Xuan Wang, Zhaoxia Li

**Affiliations:** Jiangsu Key Laboratory of Engineering Mechanics, Southeast University, Nanjing, Jiangsu, China; Virginia Tech, UNITED STATES

## Abstract

Damage in the bonding interface is a major factor that leads to the degradation of macroscopic performance of reinforced concrete (RC) structure because the damage generally results in the debonding or slipping between reinforcement and concrete. Based on hierarchical mesh methodology, a multi-scale finite element (FE) model consisting of coarse aggregate, mortar and steel rebar was established to analyze the failure process of RC structure in this paper. In order to develop the mesoscopic FE model, Monte-Carlo method was used to randomly generate the size and position of coarse aggregates; a criterion of mesh reconstruction was proposed to separate the macroscopic mesh into the mesoscopic mesh and the mesh of transitional zone; the damage constitutive relation model for concrete presenting significant difference of its tensional and compressive properties was adopted to control the damage evolution in concrete when loading; the birth-death element method was used to adaptively reform the multi-scale FE model, and finally macroscopic performance degradation of RC structure was evaluated reasonably. A example of standard RC specimen under unaxial load was performed to verify both the accuracy and efficiency of the developed FE model in analyzing failure mode of RC specimen under unaxial tension and compression. By using the developed multiscale FE model, the destruction process of a four-point bending RC beam was analyzed. The simulation results coincide well with the test results from another literature.

## 1 Introduction

From the perspective of mechanics, the degradation of bonding ability in the interface between concrete and steel rebar when loading is caused by the shear stress due to the difference of their material properties. When the shear stress reaches to a critical value, the relative slip between concrete and steel rebar along the anchorage direction is generated in the bonding interface. If the slippage is small, these two materials still can collaborate; while the debonding is generated once the slippage exceeds a certain critical value. Main interactions between concrete and reinforcement in the process of debonding include pullout effect [1–2], tension-stiffening effect [3–5] and dowel effect [6–7].

At the present, research methods for the interfacial bonding performance of the RC structure mainly include the pull-out test [8–10] and the numerical simulation [11–12]. Various bond-slip constitutive models were developed based on the long-term experimental research. After measuring and analyzing the results in pull-out tests, Miraza and Houde [13] obtained the relationship of bond stress and local slip and developed continuous curve equations. According to experimental results of double pullout tests, Kankam [14] developed the bond-slip relation equations of three different kinds of steel rebar, including smooth steel rebar, cold formed steel rebar and hot rolled steel rebar. T. P. Tassion [15] developed a hexagonal line model of local bond-slip relationships in the basis of existed experimental and simulative results. However, due to numerous factors, complicated damage mechanism and different test conditions, the above constitutive relationship models are different and there is no uniformed model of use.

The finite element method is a supplement of experimental research and has been used widely because of its convenience in research on reinforced concrete bond behavior. At present, there are two strategies to deal with the interaction between concrete and reinforcement: 1) (continuous mode) an average stress-strain relationship of concrete and reinforcement is developed to modify the constitutive relationship of concrete or reinforcement [4, 16–17]; 2) (discontinuous mode) an interface element is settled between concrete elements and steel rebar elements, in which relationships of shear stress and relative slip are considered and interactions of concrete and reinforcement is simulated [12,18–19].

From the perspective of material damage, the bonding failure of reinforced concrete is a multi-scale process that mesoscopic damage accumulates and then induces macroscopic rupture [20]. Specifically speaking, mortar hardens, dissipates heat and finally contracts in the process of concrete forming, which produces microcracks and microvoids in the interface of mortar and reinforcement. Under internal and external effects, the initial interfacial micro cracks and holes gradually nucleate, propagate, connect and then evolve into macro cracks which deteriorate the interfacial bonding performance of reinforced concrete and finally result in the debonding and failure of the component. Most researches on damage evolution in the bonding interface of reinforced concrete based on macroscopic phenomenological method. For example, Soh et al. [21] proposed a damage model for the concrete-steel interface to represent the constitutive law of the lumped interface model. Both the normal and tangential damage factors are defined as well as their evolution equations are derived. The coupling between these two damage factors is studied and finally the experiments were carried out to verify the model. Alfano et al. [22] proposed a new model that simultaneously considered interfacial damage and cohesion caused by friction effect based on Crisfield’s interfacial model [23] and coulomb's friction law respectively. According to the small deformation assumption and the plane-strain assumption, Dominguez and Ibrahlmbegovic [24] developed an inelastic constitutive relation model of bonding interface to describe bond-slip behavior of reinforced concrete.

The above models and methods focus on the macro mechanical performance of bonding interface of reinforced concrete. They don’t consider the micro/meso-structure of bonding interface, which means that they aren’t able to describe condition and transformation of damage in a microscopic or a mesoscopic level. However, concrete material is a highly heterogeneous material comprised of coarse aggregate, mortar and interfacial transition zone (ITZ) between them [25]. Random micro/mesoscopic heterogeneity plays a key role in the macroscopic mechanical properties and the failure process of reinforced concrete [20]. Therefore, in order to enhance the understanding of debonding process of reinforced concrete, it is necessary to consider the material heterogeneous structures in the mesoscopic level.

In order to overcome the shortcomings of traditional research methods, this paper developed a multiscale simulation method to research debonding process of reinforced concrete. This method presents real meso-structural morphology and random non-uniformities of RC material. First, a multi-scale finite element model based on adaptive mesh refinement algorithm was established, in which the mesoscopic damage evolution in the bonding interface was simulated. Second, the feasibility and accuracy of the developed FE model was verified by test results of concrete specimen under uniaxial tension and compression. Last but not least, the multi-scale model of a four-point bending RC beam was developed to analyze the macroscopic performance degradation and mesoscopic damage evolution process, as well as the influence of mesoscopic damage evolution on macroscopic performance degradation.

## 2 Multi-scale modeling considering the mesoscopic damage of the bonding interface

In order to study the interfacial debonding or slipping of reinforced concrete caused by mesoscopic damage, it is necessary to research the failure process in a mesoscopic level. However, the large volume of concrete structure makes the computation of mesoscopic model inefficient. Therefore, a multi-scale finite element model of the RC structure is developed in this paper, which can simulate the deterioration of the macroscopic mechanical performance of the structure and the mesoscopic damage evolution on the bonding interface at the same time. There are mainly three steps to develop the multi-scale model: 1) developing a mesoscopic model of concrete material with random coarse aggregates, 2) realizing multi-scale modeling of the RC structure by adaptive mesh encryption technology and 3) calculating the damage of each element in every analysis step to simulate damage evolution in the bonding interface.

### 2.1 Mesoscopic modeling of the bonding interface

The mesoscopic modeling of bonding interface includes the following steps: 1) determining the location and size of random coarse aggregates, 2) modeling and meshing the bonding interface (2D model) in commercial software (ANASYS in this paper), and 3) assigning constitutive relationships to the mesoscopic elements.

#### 2.1.1 Initial random mesoscopic model of the bonding interface

At the mesoscopic level, steel could be regarded as a homogeneous material, and thus the model only needs to consider the surface shape of steel rebar. However, concrete is heterogeneous and consist of two components, mortar and coarse aggregate. Therefore, the established mesoscopic model should describe both the surface shape of the steel rebar and the distribution of coarse aggregates in the mortar. The shape and the location of steel rebar could be determined by the design parameters. Meanwhile, according to the gradation or the ratio of concrete, the number, size and position of the coarse aggregates could be generated by Monte Carlo simulation.

**Fig 1:**
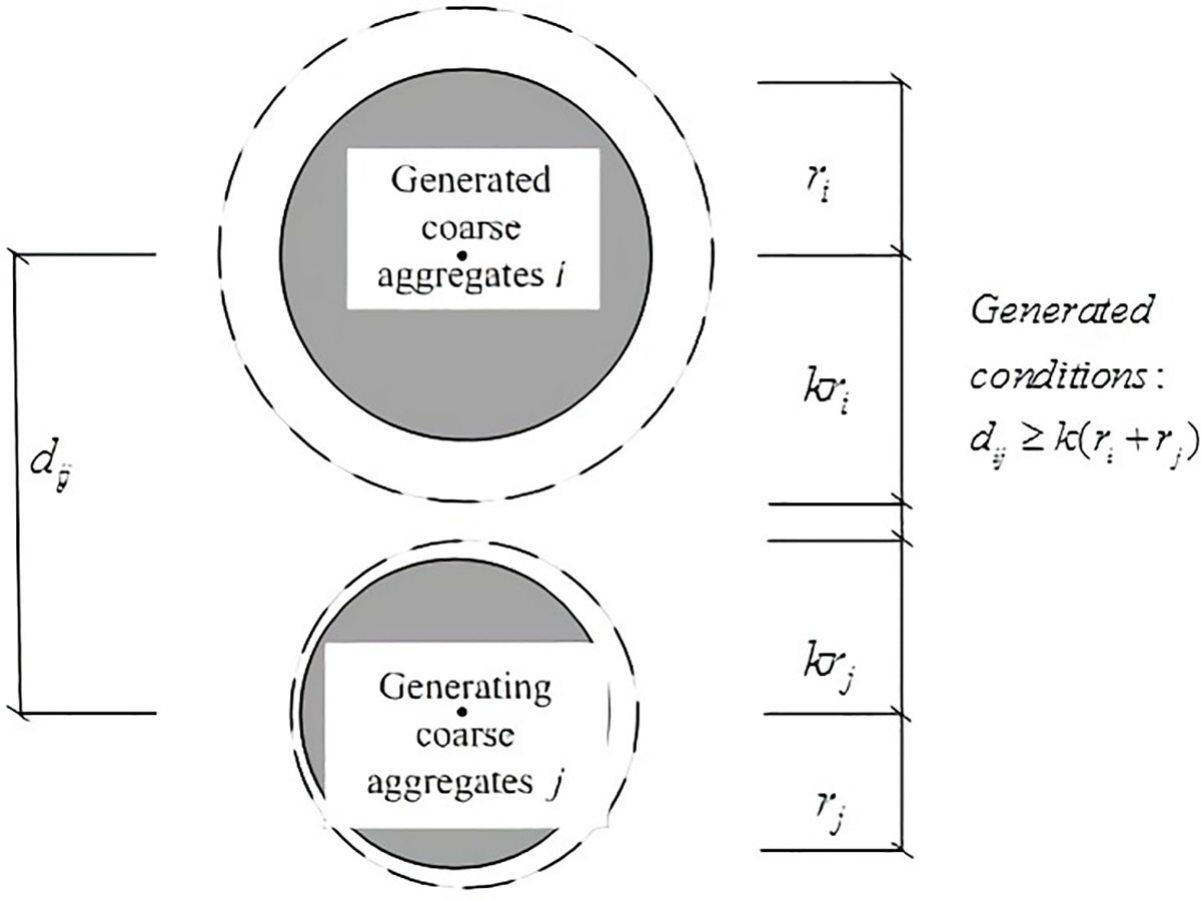
Sketch of random aggregate generation. *r*_*i*_ and *r*_*j*_ are the radii of the ith and jth aggregate, and *k* is the amplification factor.

The first step in Monte-Carlo process is to determine the maximum size of the grains, and then the coarse aggregates are generated one by one based on random centroid position and diameter within the maximum size range. The coarse aggregate isn’t allowed to locate near the generated coarse aggregates and steel rebar. When total area of the aggregates whose diameters are within the current size range meets the requirement, the coarse aggregates within the next size range are generated sequentially until all the coarse aggregates are generated. The order of the generation process is from the maximum size range to the minimum size range. The schematic diagram of random aggregate generation process is shown in Fig 1, in which *r*_*i*_ and *r*_*j*_ are the radii of the ith and jth aggregate, and *k* is the amplification factor.

**Fig 2:**
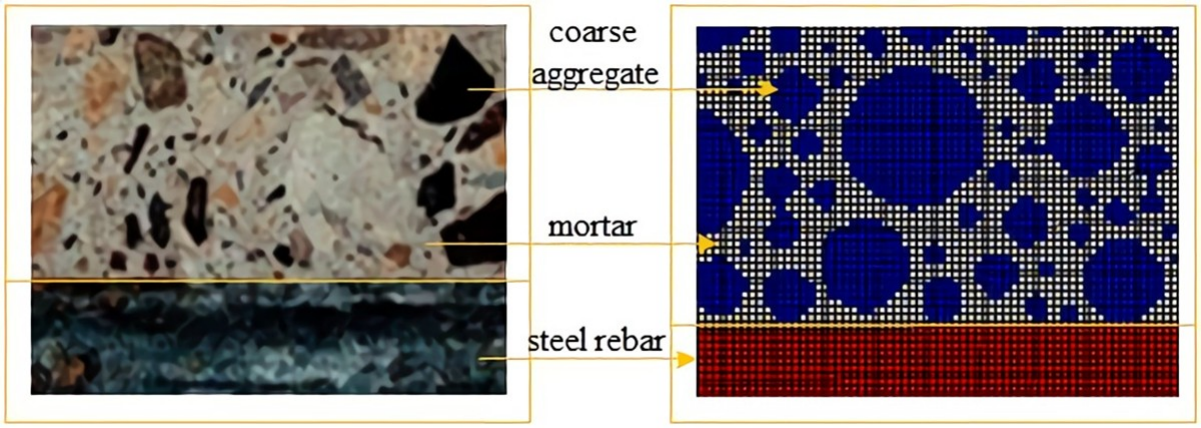
Mesoscopic bonding interface of reinforced concrete. a) Image of the real bonding interface zone. b) Mesoscopic finite element model of the bonding interface. The blue elements are coarse aggregates, the gray elements are mortar and the red elements are steel rebar. A grid size of 1mm*1mm is taken to ensure the homogeneity of structural interior at mesoscopic level.

The mix ratio of concrete chosen in this paper is that the coarse aggregate accounts for about 45% of the total volume of concrete, including big grains whose diameters range from 20mm to 40mm and small grains whose diameters range from 5mm to 20mm [26]. The mass ratio of these two grains is 55:45 [26]. According to ref [27], the amplification factor k is set as 1.1 to ensure that grains completely are located on the unoccupied region. The Monte-Carlo algorithm is compiled in MATLAB software and then produced data are inputted into ANASYS to establish initial mesoscopic model of the bonding interface as shown in Fig 2. Fig 2A) is the actual image of bonding interface, and Fig 2B is a mesoscopic finite element model of the bonding interface, in which the blue elements are coarse aggregates, the gray elements are mortar and the red elements are steel rebar. Given the minimum grain size of coarse aggregates is about 5mm and the width and height of the rib of steel rebar is 1-2mm, a grid size of 1mm*1mm is taken to ensure the homogeneity of structural interior at mesoscopic level.

#### 2.1.2 Constitutive relationship of mesoscopic elements

Linear elastic constitutive relationship is used to describe the mechanical property of steel, and the constitutive relationship with the damage variable *D* is used for the mortar and coarse aggregates.

Steel is modeled with linear constitutive relation, and the stress-strain equation is as follows:

$$\sigma_{s}=E_{s}\varepsilon_{s}$$ (1)

where *E*_*s*_ is the elastic module of the steel, which is 210GPa [26] in the drawing test.

**Fig 3:**
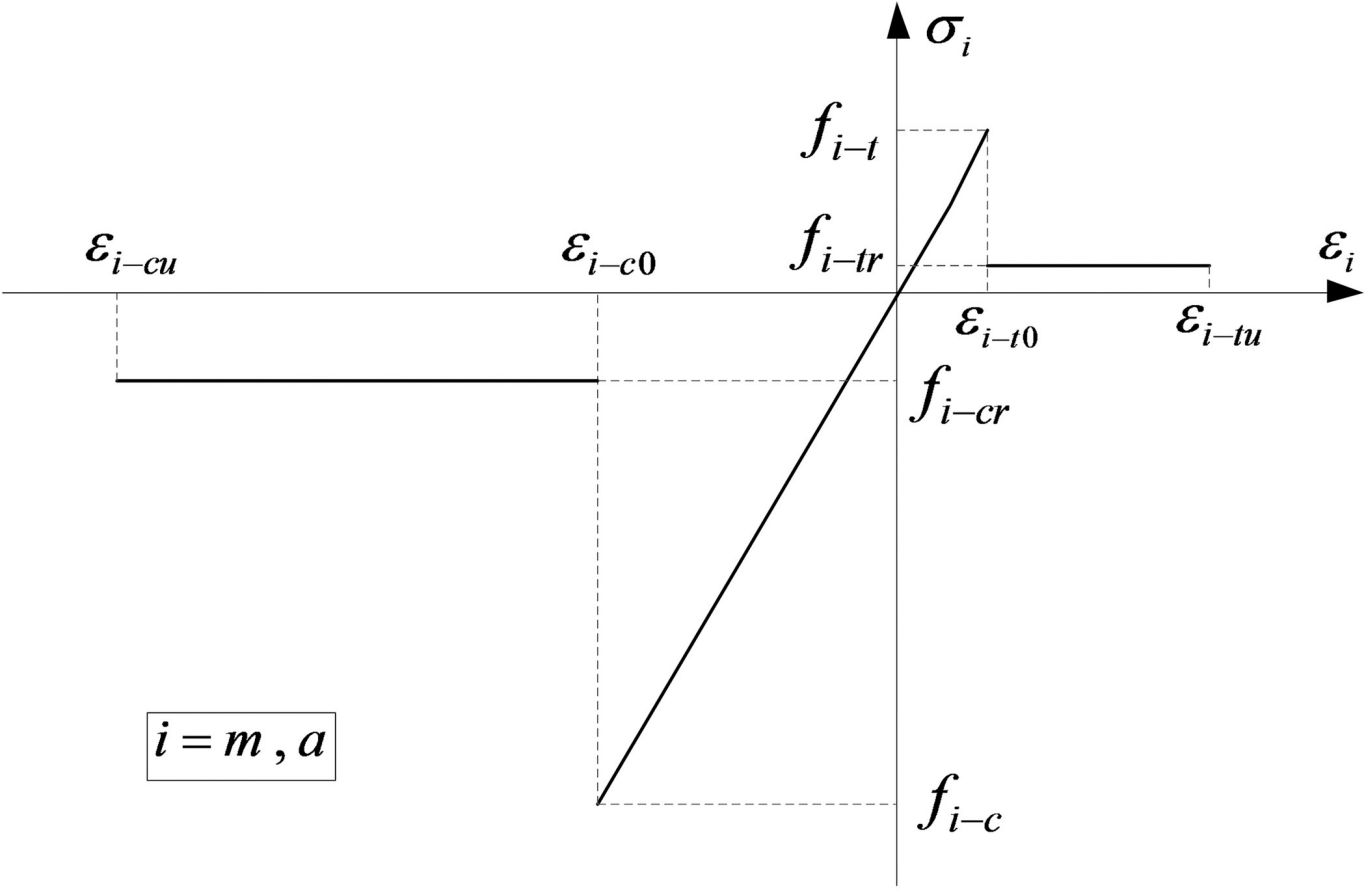
Constitutive relation of mortar and coarse aggregate [28]. *ε*_*i*−*t*0_ is the tensile strain threshold for damage evolution and *ε*_*i*−*tu*_ is the tensile strain limit. *f*_*i*−*t*_ is the uniaxial tensile strength and *f*_*i*−*tr*_ is the residual tensile strength after damage initiation. *ε*_*i*−*c*0_ is the compressive strain threshold and *ε*_*i*−*cu*_ is the compressive strain limit. *f*_*i*−*c*_ is the uniaxial compressive strength and *f*_*i*−*cr*_ is the residual compressive strength after damage initiation.

The constitutive relationships of mortar and coarse aggregates are shown in Fig 3, in which *m* and *a* refer to the mortar and coarse aggregates respectively. The constitutive equation is as follows:

$$\sigma_{i}=E_{i}(1-D_{i})\varepsilon_{i},\;i=m,a$$ (2)

where *E*_*m*_ and *E*_*a*_ are the initial elastic modulus of mortar and coarse aggregates respectively. Based on the uniaxial compression test [28] and the proportioning scheme of the common concrete, *E*_*m*_ is 22.2GPa and *E*_*a*_ is 75GPa. *E*_*c*_, the elastic modulus of the concrete, is taken as 31.5GPa

The damage variables of mortar and coarse aggregates are denoted as *D*_*m*_ and *D*_*a*_ respectively. The damage resulted from the previous loading increment step is introduced into the elastic modulus of the later loading increment step to simulate the damage evolution of the mesoscopic RC structure. *D*_*R*_ refers to the damage zone where *D*_*m*_>0 or *D*_*a*_>0. The mesoscopic damage evolution is characterized by both the extension of damage area and the increase of damage value of mesoscopic mortar elements. The mesoscopic damage evolution conditions will lead to the macroscopic performance deterioration, including the area of damage zone, the direction of damage propagation, the extension rate, etc.

The damage variable *D*_*i*_(*i* =*m*,*a*) under uniaxial tension is expressed as follows:

$$D_{i}=\left\{ \begin{array}{ll} 0 & 0<\varepsilon_{i}<\varepsilon_{i-t0}\\ 1-\frac{\lambda_{i-t}\varepsilon_{i-t0}}{\varepsilon_{i}} & \varepsilon_{i-t0}\leq \varepsilon_{i}<\varepsilon_{i-tu}\\ 1 & \varepsilon_{i}\geq \varepsilon_{i-tu} \end{array}\right.,\;i=m,a$$ (3)

where *ε*_*i*−*t*0_ is the tensile strain threshold for damage evolution and *ε*_*i*−*tu*_ is the tensile strain limit. The residual tensile strength coefficient *λ*_*i*−*t*_ is defined by the relationship: *f*_*i*−*tr*_ = *f*_*i*−*t*_*λ*_*i*−*t*_, where *f*_*i*−*t*_ is the uniaxial tensile strength and *f*_*i*−*tr*_ is the residual tensile strength after damage initiation.

The damage variable *D*_*i*_(*i* =*m*,*a*) under uniaxial compression is expressed as follows:

$$D_{i}=\left\{ \begin{array}{ll} 0 & 0>\varepsilon_{i}>\varepsilon_{i-c0}\\ 1-\frac{\lambda_{i-c}\varepsilon_{i-c0}}{\varepsilon_{i}} & \varepsilon_{i-c0}\geq \varepsilon_{i}>\varepsilon_{i-cu}\\ 1 & \varepsilon_{i-cu}\geq \varepsilon_{i} \end{array}\right.,\;i=m,a$$ (4)

where *ε*_*i*−*c*0_ is the compressive strain threshold and *ε*_*i*−*cu*_ is the compressive strain limit. The residual compressive strength coefficient *λ*_*i*−*c*_ is defined by the relationship: *f*_*i*−*cr*_ = *f*_*i*−*c*_*λ*_*i*−*c*_, where *f*_*i*−*c*_ is the uniaxial compressive strength and *f*_*i*−*cr*_ is the residual compressive strength after damage initiation.

**Fig 4:**
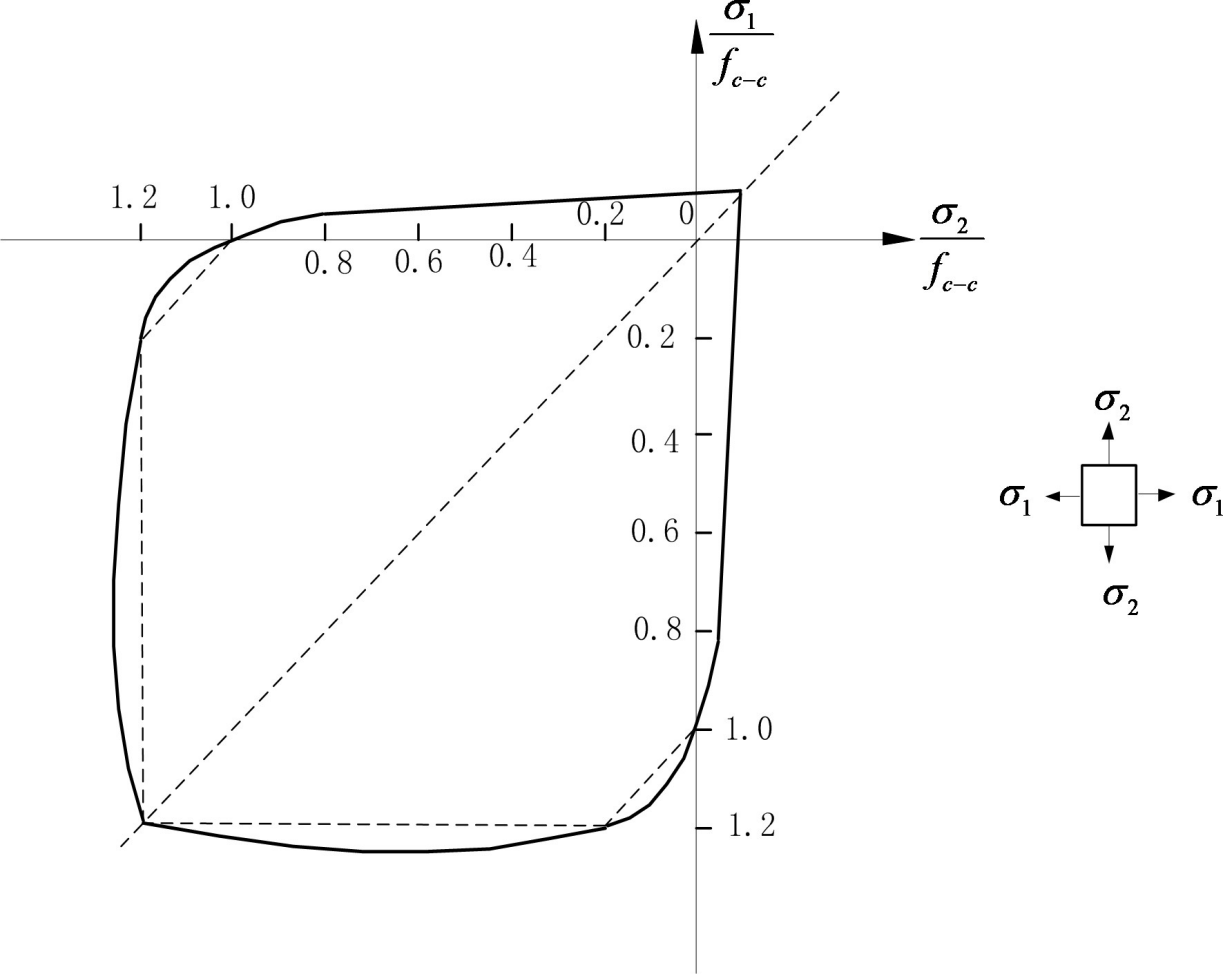
Strength criterion of concrete under biaxial stress state [29]. *σ*_1_ and *σ*_2_ are the biaxial stress value. *f*_*c*−*c*_ is uniaxial compressive strength of concrete.

Because mortar and coarse aggregate are brittle materials, their mechanical properties are similar to that of concrete. Therefore, the strength criterions of mortar and coarse aggregates are also similar to that of concrete under biaxial stress, as shown in Fig 4 [29]. The characteristics of brittle materials under biaxial stress state could be summarized as follows: 1) the failure modes under biaxial compression stress and compression-shear stress are similar to that under uniaxial compression stress, and the failure modes under biaxial tension stress and tension-shear stress are similar to that under uniaxial tension stress; 2) the tensile strength under biaxial tension stress and tension-shear stress is almost the same as that under uniaxial tension stress; 3) the compressive strength under biaxial compression stress is greater than that under uniaxial compression stress, and the compressive strength under compressive-shear stress is smaller than that under uniaxial compression stress.

According to the characteristic 1), the damage equations of mortar and coarse aggregate under biaxial stress could be obtained from the Eq (3) and Eq (4). When *σ*_1_>*σ*_2_>0, regarding *σ*_1_ as the principal stress, *D*_*m*_ and *D*_*a*_ could be calculated by Eq (3). When *σ*_2_<*σ*_1_<0, regarding *σ*_2_ as the principal stress, *D*_*m*_ and *D*_*a*_ could be calculated by Eq (4). When *σ*_1_>0 and *σ*_2_<0, the calculation of damage value should be judged by *k* = |*σ*_1_/*σ*_2_| firstly. When *k* is larger than 0.17, *D*_*m*_ and *D*_*a*_ could be calculated by Eq (3). When *k* is less than 0.17, *D*_*m*_ and *D*_*a*_ could be calculated by Eq (4).

According to the characteristics 2), the maximum tensile strain criterion could be used to determine the tensile strain threshold *ε*_*i*−*t*0_ under uniaxial tension. According to the proportioning scheme of common concrete [28], when *E*_*m*_ is 22.2GPa, the corresponding *f*_*m*−*t*_ is 4MPa, and the tensile strain threshold of mortar *ε*_*m*−*t*0_, which represents the ratio of *f*_*m*−*t*_ to *E*_*m*_, is equal to 0.00018. *ε*_*m*−*tu*_ is two times as *ε*_*m*−*t*0_ and equals 0.00036. Because the mortar is more brittle under uniaxial tension than under biaxial tension, *λ*_*m*−*t*_ could be taken as 0.3. When *E*_*a*_ is 22.2GPa, the corresponding *f*_*a*−*t*_ is 15MPa. The tensile strain threshold of coarse aggregate *ε*_*a*−*t*0_, which represents the ratio of *f*_*a*−*t*_ to *E*_*a*_, is equal to 0.002. *ε*_*a*−*tu*_ is two times as *ε*_*a*−*t*0_ and equals 0.004. Because coarse aggregate is also more brittle under uniaxial tension than under biaxial tension, *λ*_*a*−*t*_ could be taken as 0.3.

According to the characteristic 3), Mohr-Coulomb criterion could be used to determine the strain threshold *ε*_*i*−*c*0_ under uniaxial compression, which could be written as follows:

$$F_{i}=\frac{1+sin\varphi_{i}}{1-sin\varphi_{i}}\sigma_{1}-\sigma_{2}\geq f_{i-c},\;i=m,a$$ (5)

where *φ*_*i*_ is the friction angle. According to the proportioning scheme of common concrete [28], when *E*_*m*_ is 22.2GPa, the corresponding *f*_*m*−*c*_ is 40MPa. The compressive strain threshold of mortar *ε*_*m*−*cu*_ is taken as 0.00036, which is ten times of *ε*_*m*−*tu*_. When subjected to uniaxial compression, mortar is not as brittle as subjected to uniaxial tension. Therefore, *λ*_*m*−*c*_ and *φ*_*m*_ could be taken as 0.7 and 30°respectively. When *E*_*a*_ is 75GPa, the corresponding *f*_*a*−*c*_ is 150MPa. The compressive strain threshold of coarse aggregate *ε*_*a*−*cu*_ can be taken as 0.004, which is ten times of *ε*_*a*−*tu*_. By the same token, because coarse aggregate is not so brittle under uniaxial compression, *λ*_*a*−*c*_ and *φ*_*a*_ can be taken as 0.7 and 30° respectively.

### 2.2 Multi-scale modeling of the RC structure

In order to improve the computational efficiency for large RC structures, refined mesh with small element size is used for the vulnerable parts, and meanwhile coarse mesh with large element size is used for the other parts of the model, i.e., a multi-scale modeling method is applied for large RC structure.

**Fig 5:**
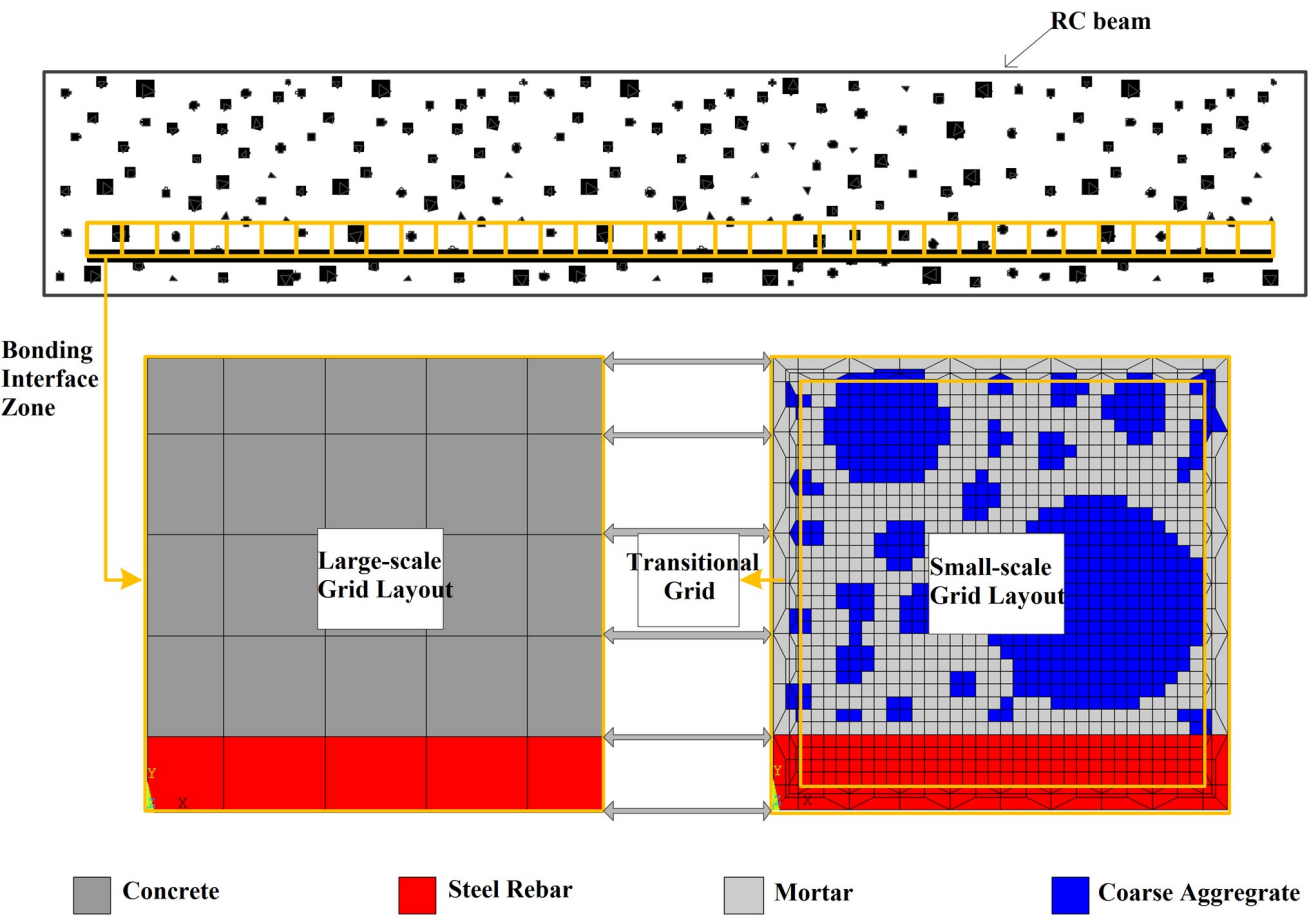
Diagram of concurrent multi-scale modeling of RC structure. The size of the local area is 36mm×36mm, in which there are 25 grids in large-scale grid layout and 1296 grids in small-scale grid layout respectively.

The established multi-scale model based on adaptive mesh encryption technology [30] is shown in Fig 5. At the initial stage, elements with a macroscopic element size are used for both steel rebar and concrete. Then stress and strain of the whole model are solved increment by increment when loading. If a macroscopic element meets the requirement of reconstruction, this element should be reconstructed with the mesoscopic mesh, including steel rebar elements, mortar elements and coarse aggregate elements with a smaller size. For instance, the local region with a dimension of 36mm×36mm shown in Fig 5 could be divided into 25 macroscopic elements. Meanwhile this region could also be divided into 1296 mesoscopic elements. Undamaged regions are still divided into macroscopic elements to improve computational efficiency. For damaged regions, the elements should be refined to ensure enough computation precision at the bonding interface and consider the influence of mesoscopic structure on damage evolution.

### 2.3 Damage evolution of the bonding interface based on the multiscale model

**Fig 6:**
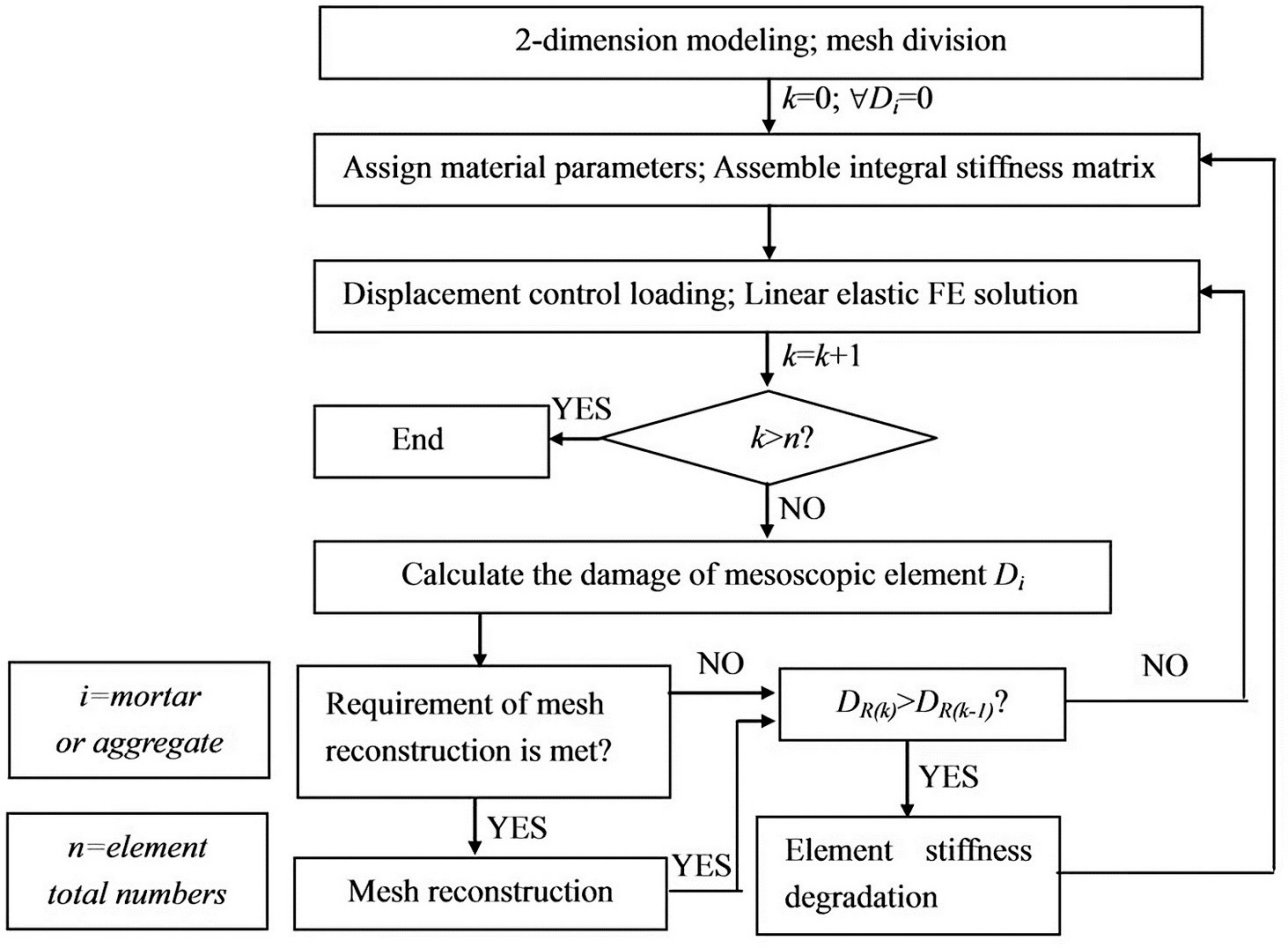
Flow of multi-scale modeling. The failure process under static action is analyzed using the multi-scale model, and the displacement load is applied increment by increment. The displacement of each increment is 0.005mm and n increments are totally loaded. At the end of each step loading, it is necessary to judge twice the damage state of the each element in the model.

The flow chart of damage evolution simulation is shown in Fig 6. External load is controlled by displacement with *n* increments, and every displacement increment is 0.005mm. The damage state of the element is judged twice in one increment.

The first judgment is to judge whether the macroscopic mesh meets the requirement of mesh reconstruction that is in line with the strain threshold criterion introduced in Section 2.1.2. *ε*_*c*−*t*0_, *f*_*m*−*c*_ and *φ*_*c*_ in Eq (5) are taken as 0.0002, 45MPa, and 30°respectively. If the concrete elements or steel rebar elements in the local region meet the requirement, the macroscopic mesh in the region is reconstructed into mesoscopic mesh and transition zone mesh and then the second judgment is conducted. If the macroscopic mesh does not meet the reconstruction criterion, the second judgment is made directly without mesoscopic mesh.

The second judgment is to judge whether the damaged zone with mesoscopic mesh and transition zone mesh increases. Firstly, the damage equation for each mesoscopic element is chosen. Then, the damage variable of the previous increment *D*_*i*−1_ and the damage variable of the current increment *D*_*i*_ are calculated. When *D*_*i*_ is not greater than *D*_*i*−1_ for all the mesoscopic elements, the damage area *D*_*R*_ remains unchanged and the calculation continues. When there exist elements in which *D*_*i*_ is greater than *D*_*i*−1_, the damage zone *D*_*R*_ increases. In this case, the elastic modulus of the damage zone is reduced and the new global stiffness matrix should be reformed before the next increment. The elastic modulus reduction process is as follows:

When *D*_*i*_ is greater than *D*_*i*−1_ but less than 1, the elastic modulus of the mesoscopic element should be reduced. The reduced elastic modulus could be expressed as:

$${\tilde{E}}_{i}=E_{i}(1-D_{i})$$ (6)

where *E*_*i*_ is the current elastic modulus and ${\tilde{E}}_{i}$ is the reduced elastic modulus.

When *D*_*i*_ reaches to 1 and is larger than *D*_*i*−1_, the mesoscopic element is “killed” by the life-and-death element method in ANSYS, i.e., the stiffness of the element is multiplied by a small factor. In the solution process, the degree of freedom of the “killed” element is constrained to prevent the singularity of the solution.

## 3 Validity of the mesoscopic damage simulation on the bonding interface

**Fig 7:**
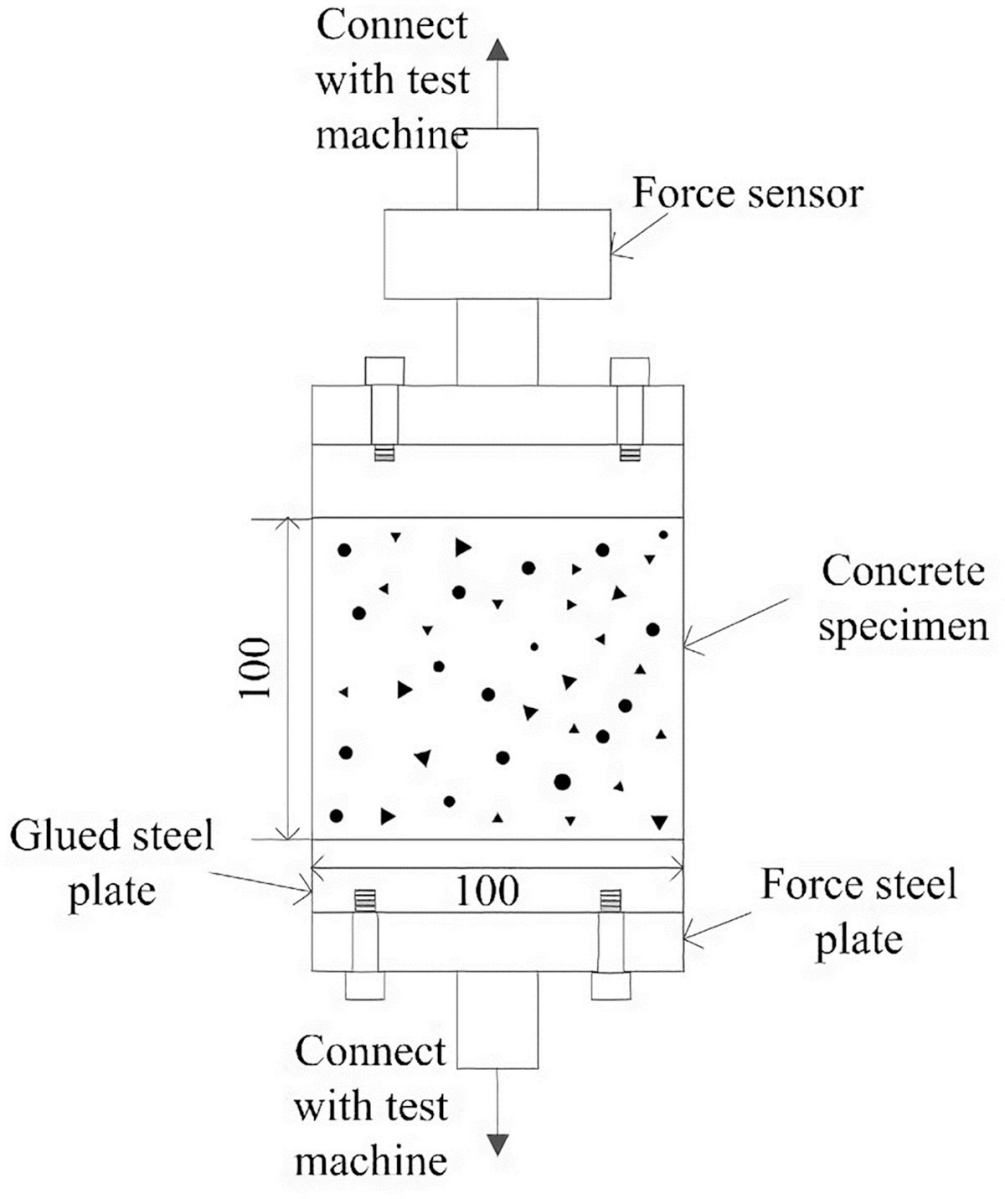
Glued double-plated concrete uniaxial tensile test device [31]. The tensile stress on the bonding interface is regarded as uniform distribution in this device.

**Fig 8:**
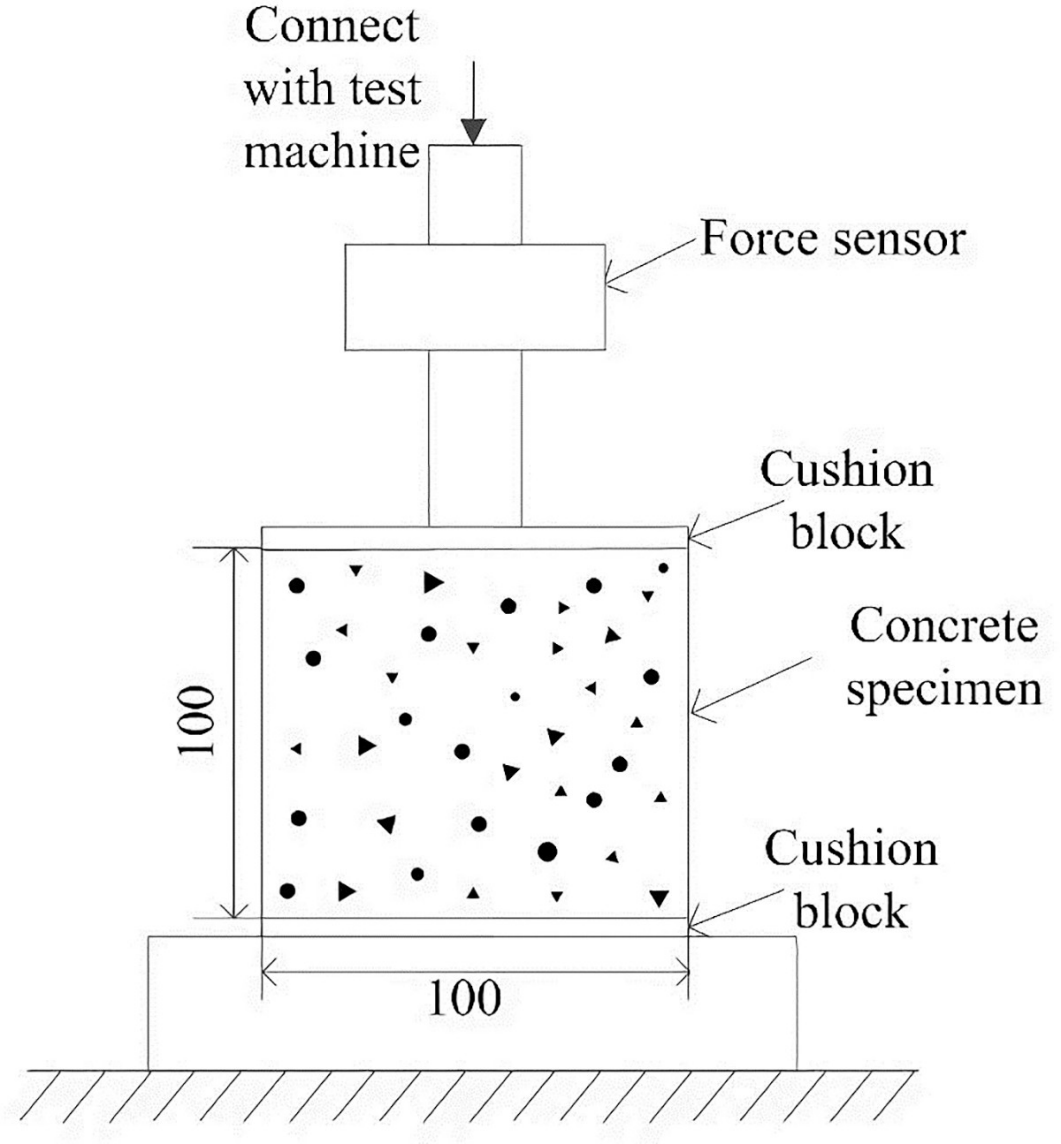
Concrete uniaxial compressive test device [31]. The compressive stress on the bonding interface is regarded as uniform distribution in this device.

In order to ensure the accuracy of the multi-scale analysis, it is necessary to make sure that the simulation at the mesoscopic scale is precise. In this paper, the mesoscopic simulation method is verified by simulating the failure process of concrete specimens subjected to uniaxial tension and compression. The glued double-plated device used in the uniaxial tensile test is shown in Fig 7, and the tensile stress on the bonding interface is regarded as uniform distribution [31]. The device used in uniaxial compressive test is shown in Fig 8, and the compressive stress on constrained ends is also regarded as uniform distribution.

**Fig 9:**
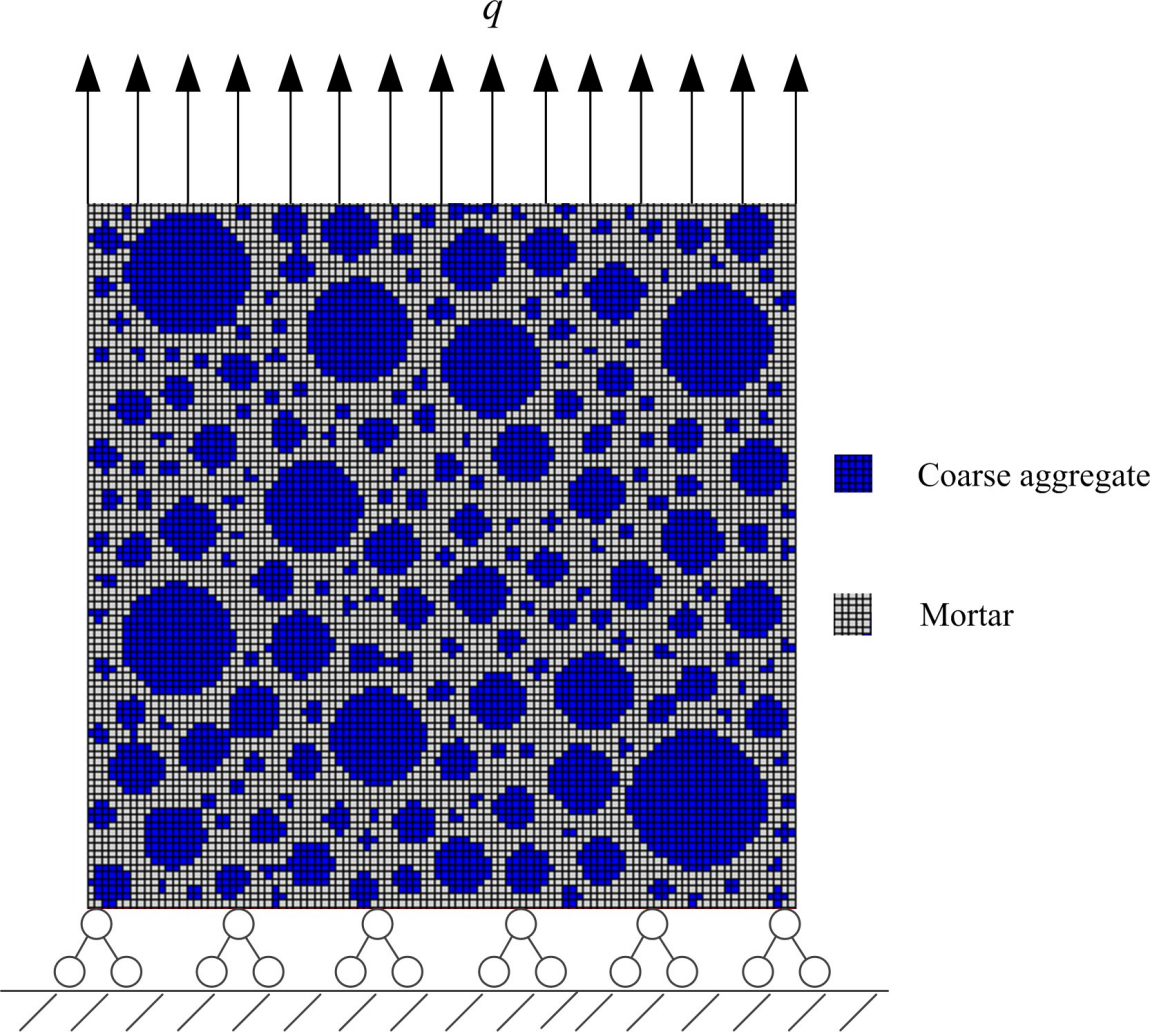
Constraints and the load of the concrete specimen. The dimension of the specimen is 100mm×100mm, and the mesh size is 1mm×1mm. The top of the concrete block is uniformly loaded, the bottom is fixed and the lateral sides are free. In uniaxial tensile process, the pressure q is positive, while in uniaxial compressive process, q is negative.

According to the test conditions above, the mesoscopic model of concrete is developed as shown in Fig 9. The dimension of the specimen is 100mm×100mm, and the mesh size is 1mm×1mm. The top of the concrete block is uniformly loaded, and the bottom is fixed and the lateral sides are free. In uniaxial tensile process, the pressure *q* is positive, while in uniaxial compressive process, *q* is negative.

**Fig 10:**
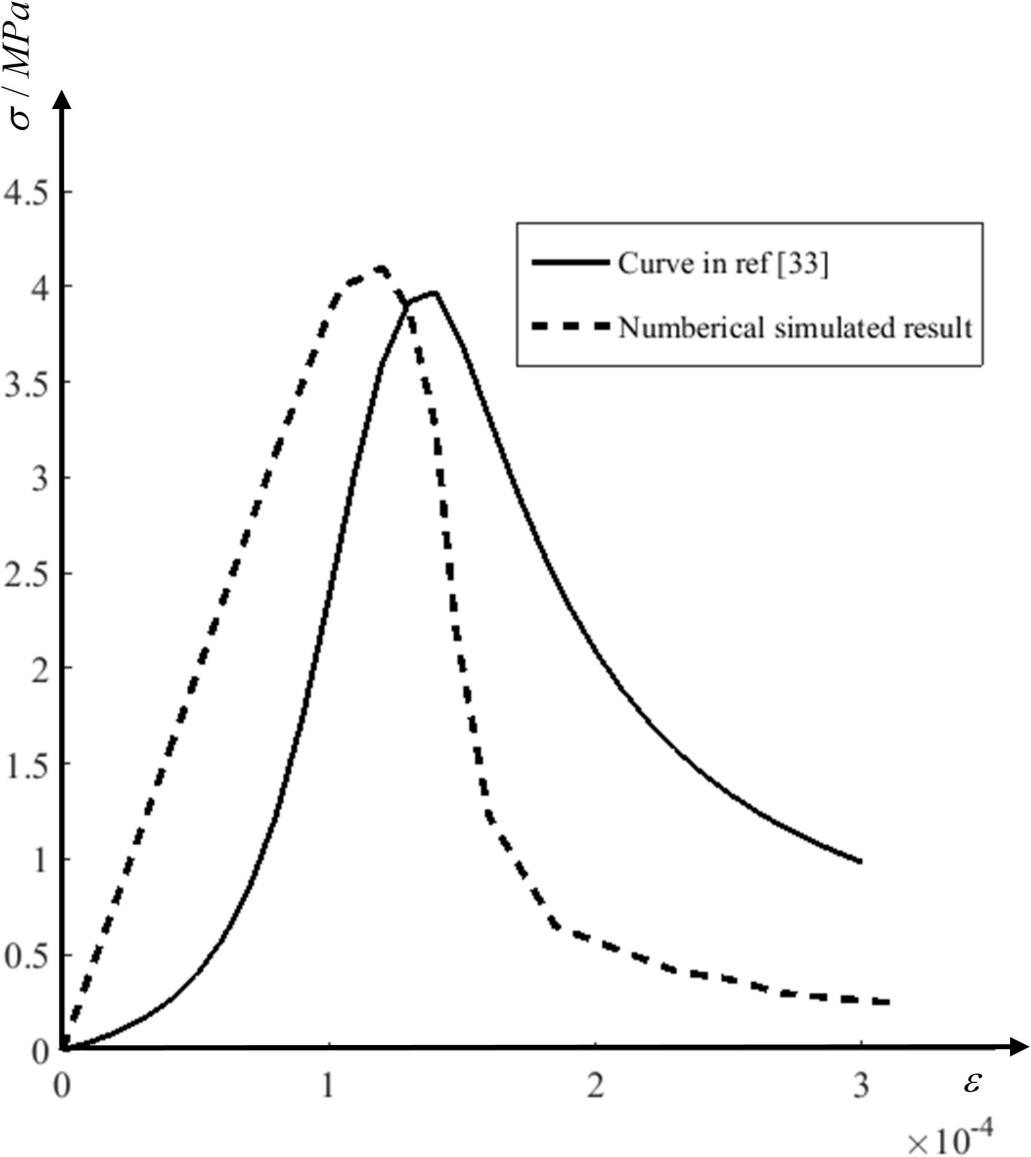
Stress-strain curves of concrete under uniaxial tension. The numerical result obtains from the FE model in Fig 9.

The stress-strain curves subjected to uniaxial tension given by the design specification [32] and obtained by the proposed multi-scale method is shown in Fig 10. It could be seen that the stress increases linearly with strain in the initial loading phase. When strain reaches 0.002, stress starts to increase nonlinearly. After the stress reaches the peak value, the curves decrease rapidly. The uniaxial tensile strength obtained from simulation is about 4MPa. The trends of the two curves are basically consistent. Considering the randomization of coarse aggregates, the gap between two curves is reasonable in Fig 10.

**Fig 11:**
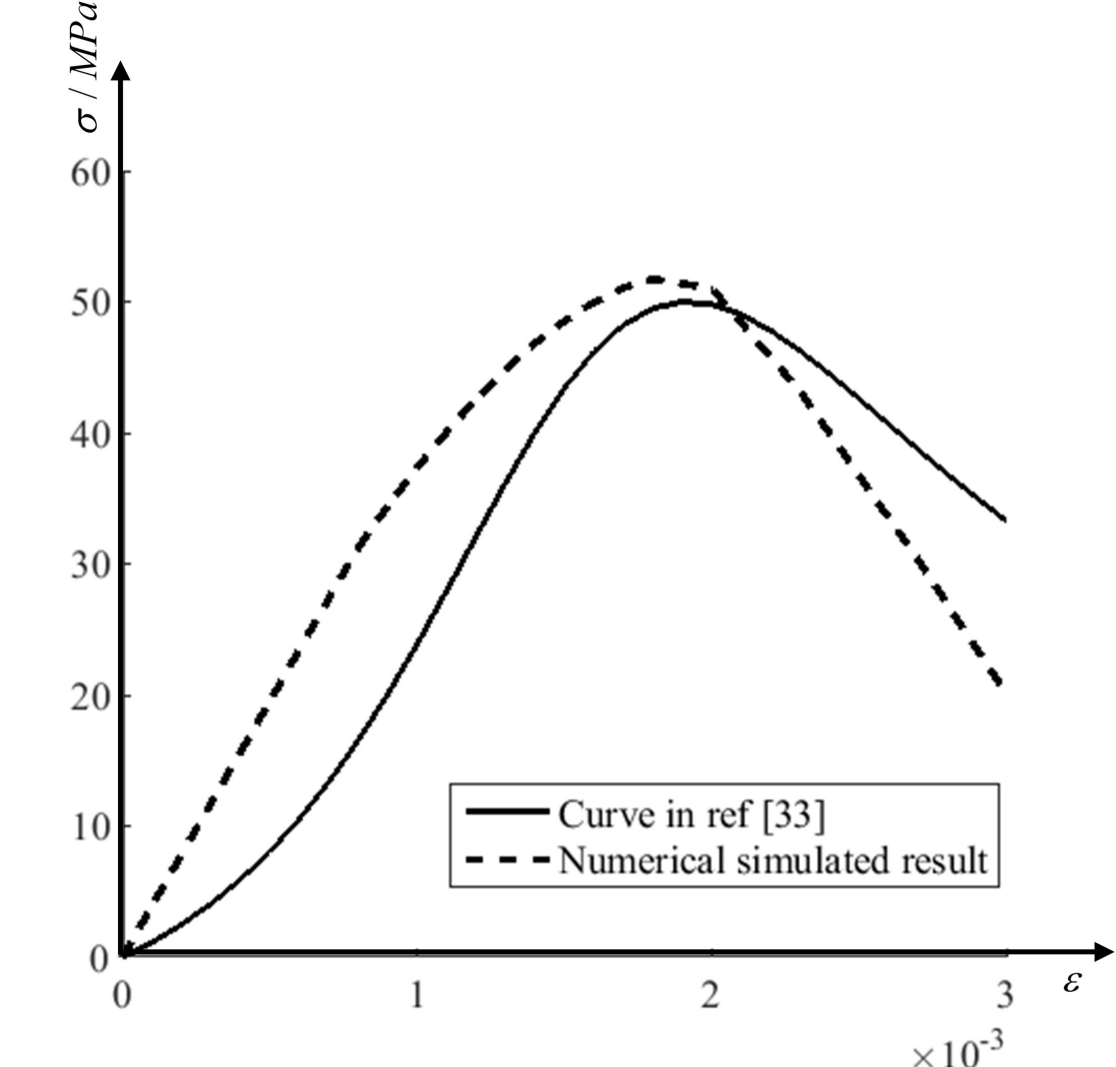
Stress-strain curves of concrete under uniaxial compression. The numerical result obtained from the FE model in Fig 9.

The stress-strain curves of concrete subjected to the uniaxial compression given by the design specification [31] and obtained from mesoscopic simulation are shown in Fig 11. It could be seen that the trends of the two curves are similar. The uniaxial compressive strength obtained from simulation is about 50MPa, 10 times of uniaxial tensile strength, which is also in line with the curve in ref [32]. Considering the randomization of coarse aggregates, the gap between two curves is also reasonable in Fig 11.

**Fig 12:**
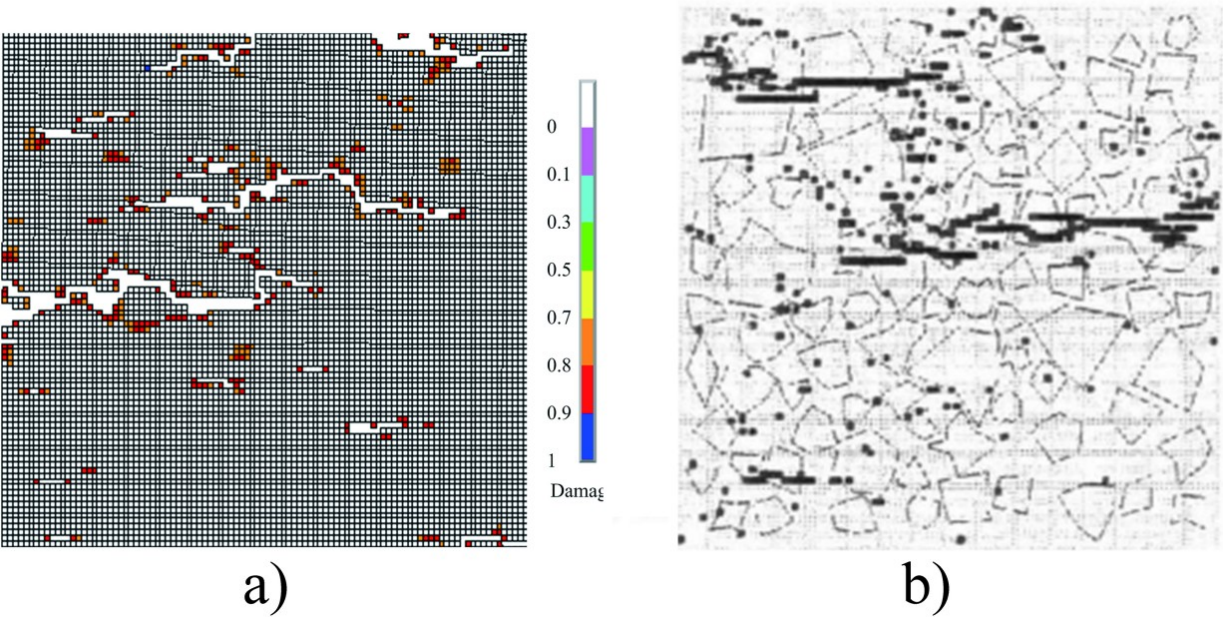
Failure modes of concrete under uniaxial tension. a) The numerical result obtained from the FE model in Fig 9. b) The result given in the literature [33].

The failure modes of concrete under uniaxial tension obtained from simulation and given in literature [33] are shown in Fig 12. It could be seen that there is more than one macro-crack perpendicular to the loading direction within the specimen, and the main crack is located in the middle of the specimen, and the two failure modes are similar.

**Fig 13:**
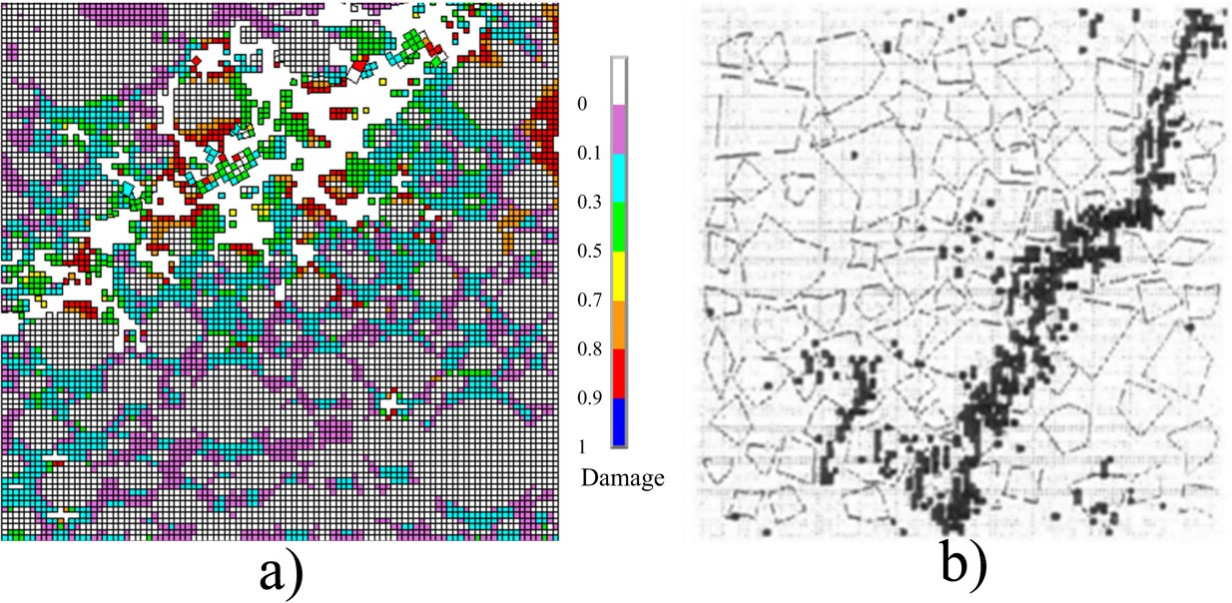
Failure mode of concrete under uniaxial compression. a) The numerical result obtained from the model in Fig 9. b) The result given in the literature [33].

The failure modes of concrete subjected to uniaxial compression are shown in Fig 13. Fig 13A) shows the damage contour obtained from simulation and Fig 13B) shows the contour given in literature [33]. It could be seen that the two damage contours are similar, and an obvious shear fracture zone could be observed.

In summary, the stress-strain curves and failure modes of concrete under uniaxial tension and compression could be obtained by the proposed mesoscopic simulation method in Section 2. They are both consistent with the design specification and the literatures. It could be seen that the proposed mesoscopic simulation method is valid and could be used in further research on macroscopic performance deterioration and mesoscopic damage evolution of RC structures.

## 4 Multi-scale analysis of four-point bending RC beam

**Fig 14:**
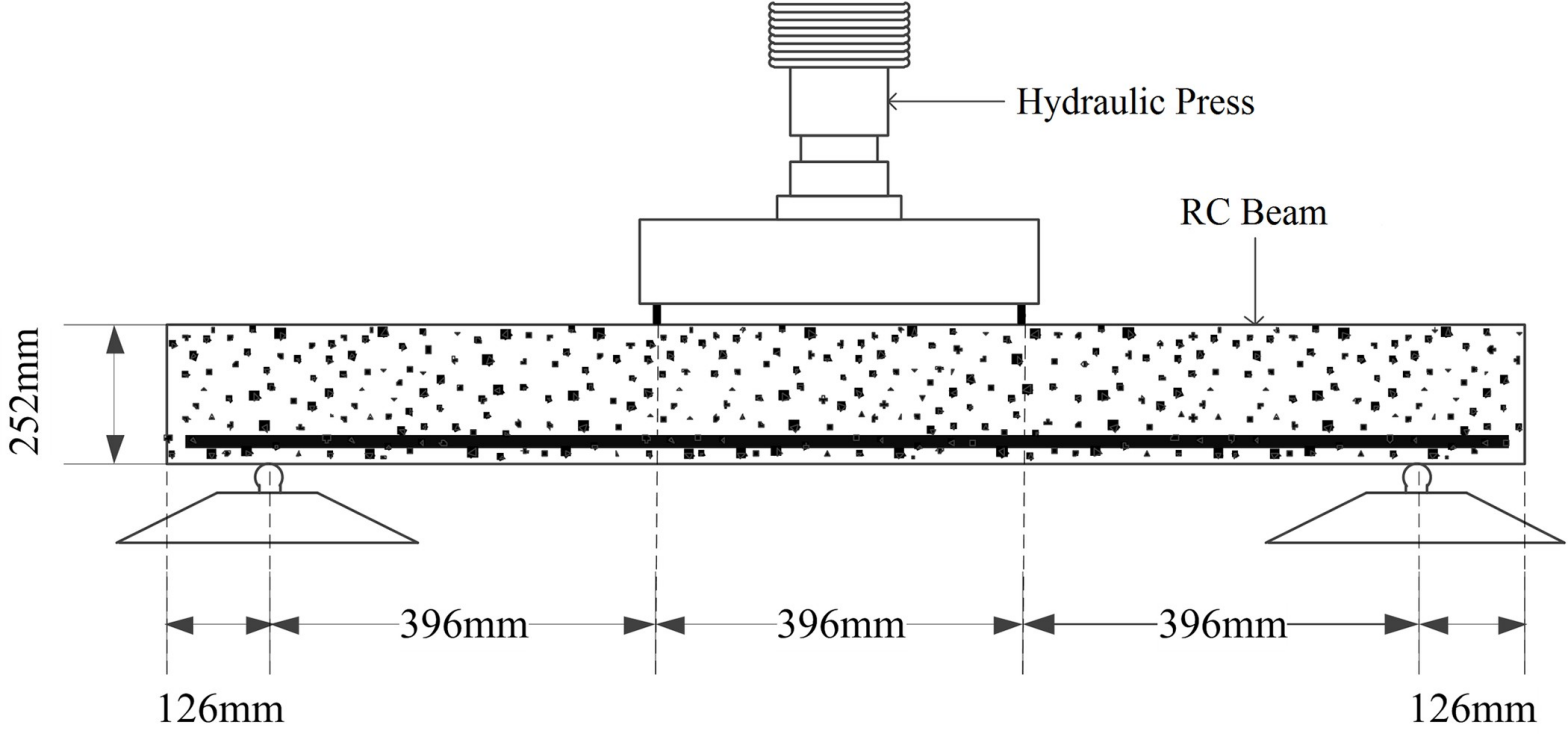
Test device and four points bending RC beam [34].

In this section, the multi-scale modeling method proposed in Section 2, in which the mesoscopic damage on the bonding interface is considered, is applied to simulate the failure process of a four-point bending RC beam. The effect of mesoscopic damage evolution on macroscopic performance of the beam is investigated, and the simulation method is verified. The test device and the size of the specimen are shown in Fig 14 [34].

**Fig 15:**
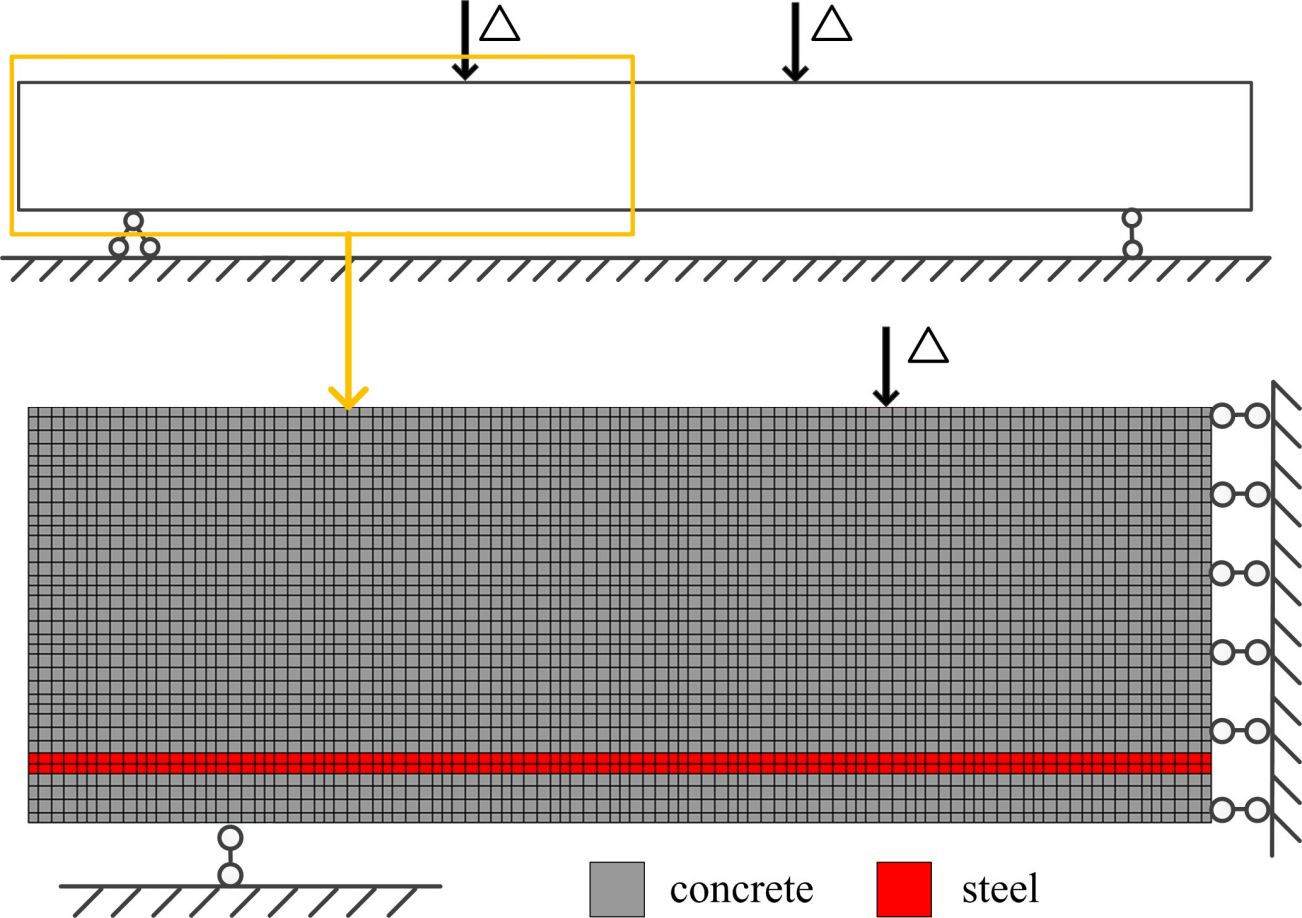
Four-point bending RC beam model. The half of the beam in Fig 14 is modeled because of symmetry. The horizontal displacement of mid-span section of the FE model is constrained to zero.

The structural shape, boundary conditions and external loads are symmetrical, and the axis of symmetry is the mid-span section. Therefore, half of the beam is modeled for simplification as shown in Fig 15. The horizontal displacement of mid-span section is constrained to zero. Material parameters of the elements take the default values as stated in the previous sections.

### 4.1 Macro performance deterioration of RC beam

**Fig 16:**
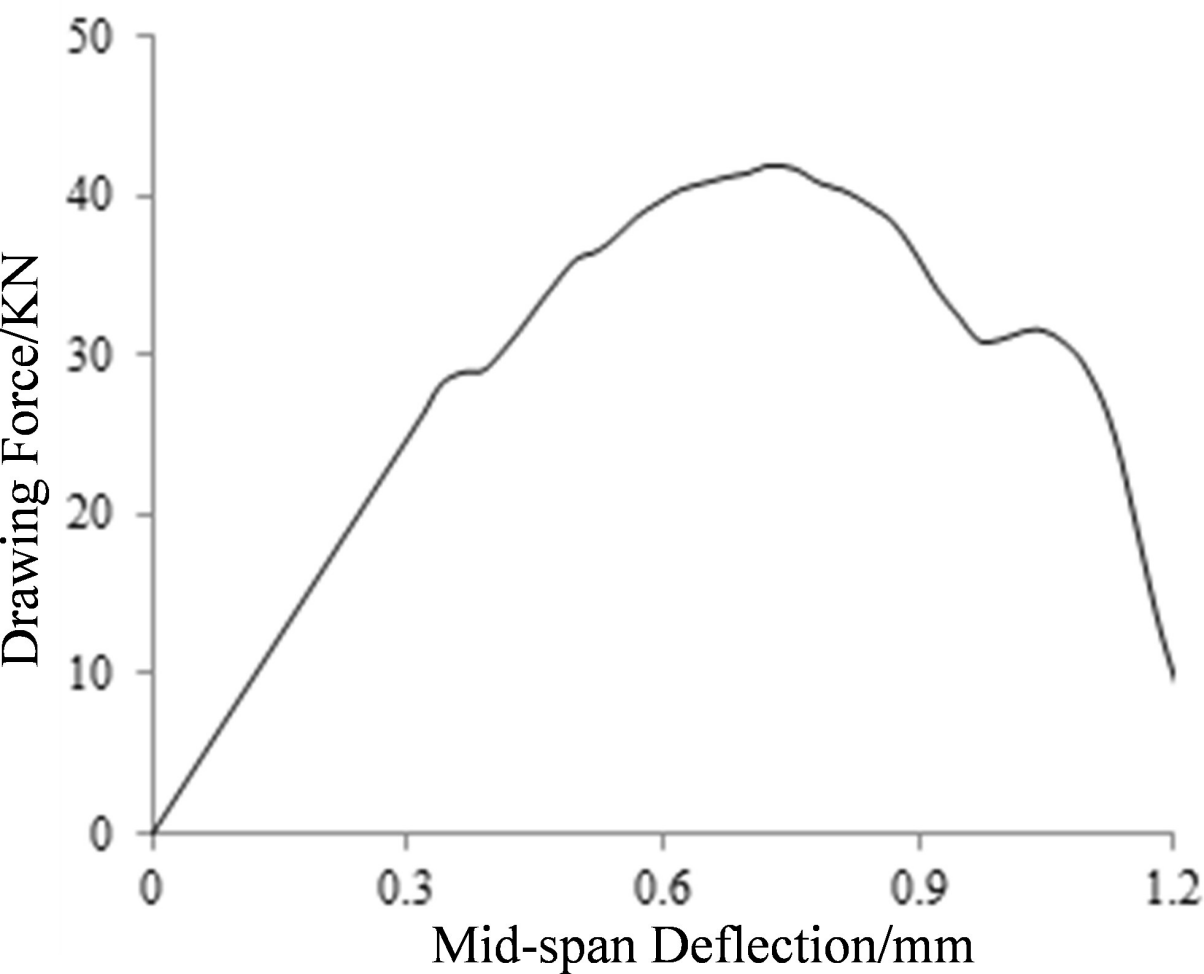
The relationship between the drawing force and mid-span deflection.

The relationship between the drawing force (reaction force of the point where displacement boundary condition is applied) and the deflection of mid-span is shown in Fig 16.

It could be seen that the drawing force increases linearly with the deflection in the initial stage. When reaching 70% of the peak value, the drawing force starts to increase nonlinearly. After reaching the peak value, the drawing force shows a downward trend.

**Fig 17:**
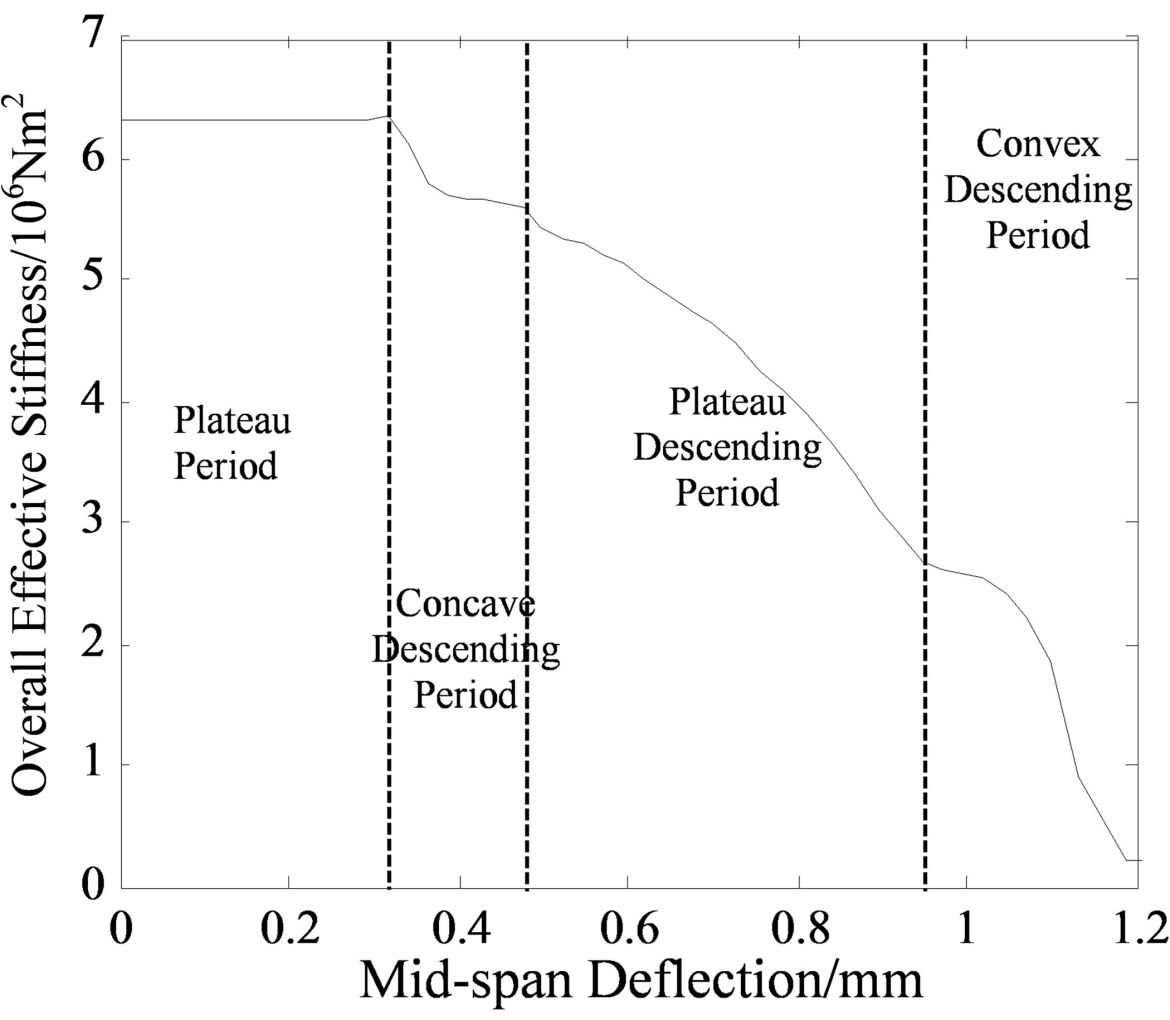
The relationship between the overall effective stiffness and mid-span deflection. The overall effective stiffness $\tilde{B}$ is defined as the product of the bending moment *M* of the portion subjected to pure bending and the mean curvature *φ* of the beam axis. The curve of overall effective stiffness can be divided into four stages, including steady increasing period, concave descending period, steady descending period and convex descending period.

The overall effective stiffness $\tilde{B}$ could be used to measure the macroscopic performance of beam, which is defined as the product of the bending moment *M* of the portion subjected to pure bending and the mean curvature *φ* of the beam axis. The relationship between the overall effective stiffness and mid-span deflection is shown in Fig 17.

It could be seen that the curve in Fig 17 can be divided into four stages: 1) Steady increasing period, bending resistance of the beam is not degraded; 2) Concave descending period, the overall effective stiffness decreases rapidly and then keeps steady again; 3) Steady descending period, the bending performance of the beam gradually decreases; 4. Convex descending period, the bending performance of the beam decreases rapidly until failure.

Assume that the mid-span deflection caused by the nominal bending moment in the damaged beam is equal to that caused by an effective bending moment in the undamaged beam, i.e.

$$f=\frac{\tilde{M}}{B}=\frac{M}{\tilde{B}}\Rightarrow f=\frac{M}{(1-D)B}$$ (7)

where *f* is the mid-span deflection, $\tilde{M}$ is the effective bending moment, *M* is the nominal bending moment, *B* is the initial overall stiffness and $\tilde{B}$ is the effective overall stiffness. *D* is defined as the macroscopic damage variable of the beam, which could be denoted by *D*_mac*ro*−*B*_ as:

$$D_{macro-B}=\frac{B-\tilde{B}}{B}$$ (8)

**Fig 18:**
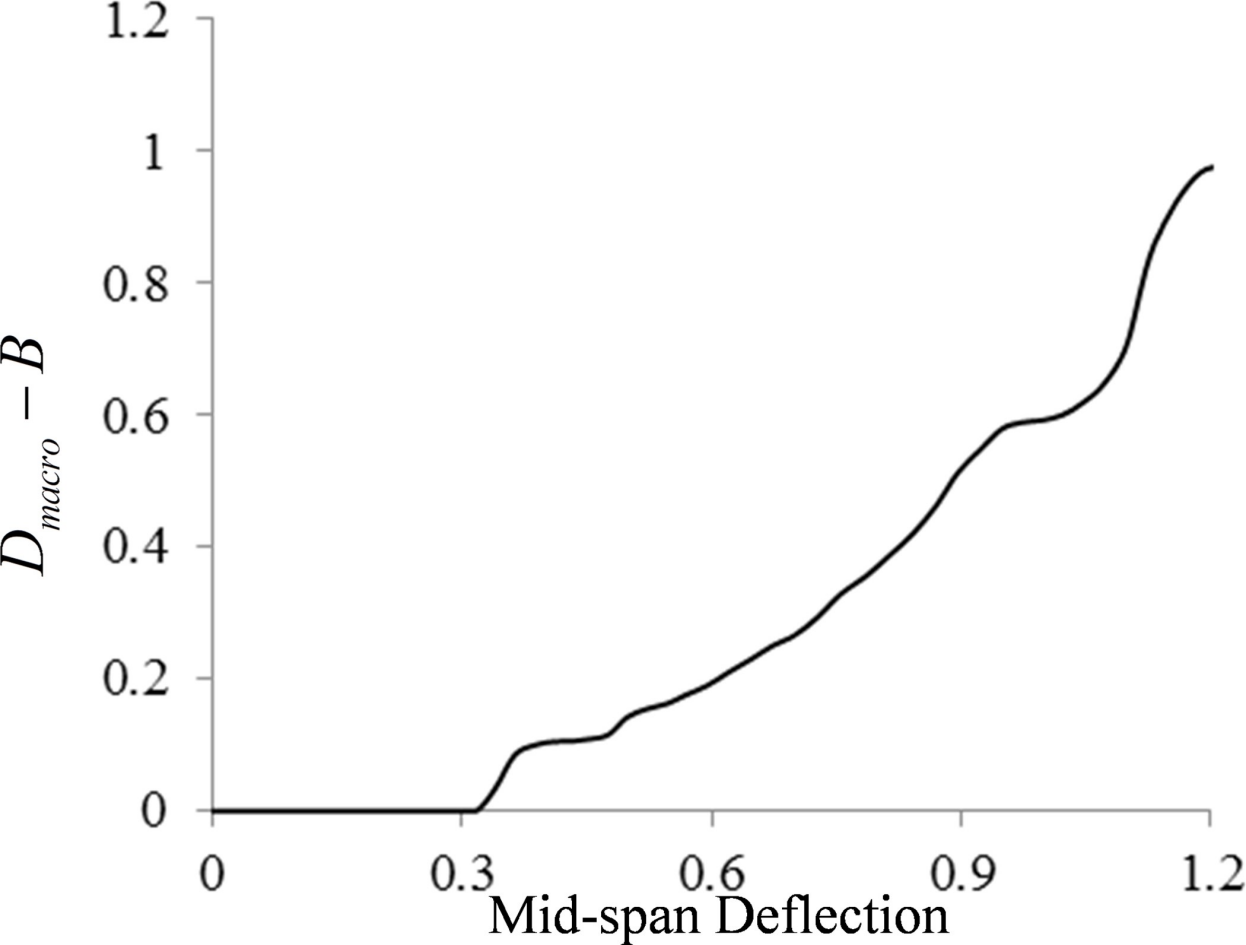
The relationship of macro damage and mid-span deflection in the four-point bending beam. *D*_mac*ro*−*B*_ is defined as the macroscopic damage variable of the beam, and its calculation method is as shown in Eq (8).

When $\tilde{B}$ is equal to *B*, *D*_*macro*−*B*_ is equal to zero, which means that there is no damage in the beam. When $\tilde{B}$ is equal to zero, *D*_*macro*−*B*_ is equal to 1 and the beam has been damaged completely. When 0<*D*_*macro*−*B*_<1, the damage is evolving gradually. The relationship between *D*_*macro*−*B*_ and mid-span deflection *f* is shown in Fig 18.

The trend of macroscopic damage evolution is opposite to that of the deterioration process of global effective stiffness, which could be divided into four stages: 1) Plateau period, the macroscopic damage is 0; 2) Concave growth period, the macroscopic damage suddenly increases to 0.1 and then tends to be steady; 3) Steady growth period, macroscopic damage increased steadily to 0.6; 4) Convex growth period, the macroscopic damage rapidly increases to 1, and the failure of the beam occurs.

### 4.2 Mesoscopic damage evolution process of RC beam

The deterioration process of bending performance of RC beam in section 4.1 is due to the mesoscopic damage evolution. By analyzing the mesoscopic damage states of the beam at different stages of the overall effective stiffness curve, the relationship between mesoscopic damage evolution and macroscopic performance degradation is studied.

**Fig 19:**
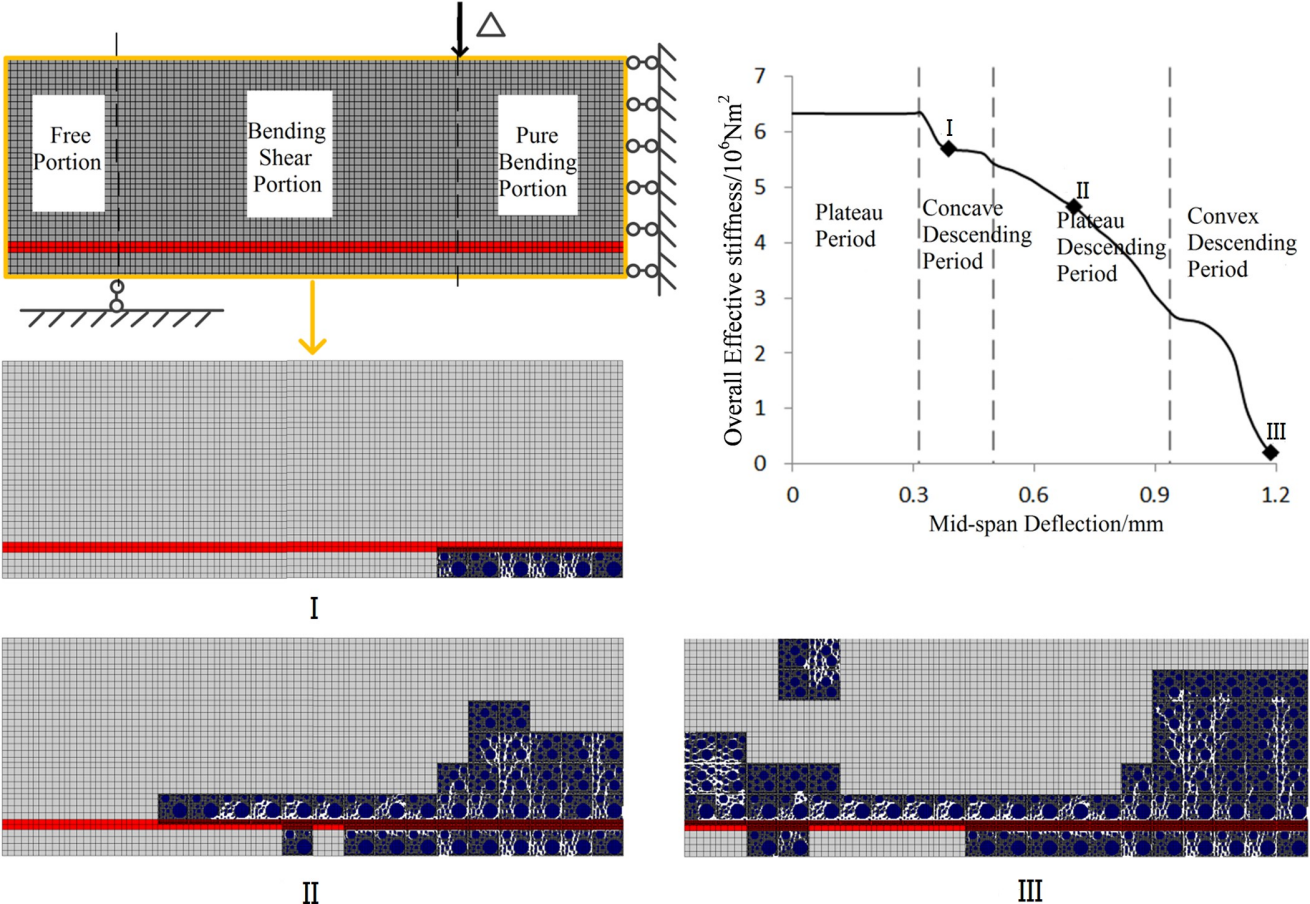
Mesoscopic-damage evolution process of the four-point bending beam. There are three damage states of I, II and III, which correspond to the points I, II and III that are located on concave descending period, steady descending period and convex descending period of the curve of overall effective stiffness respectively.

**Fig 20:**
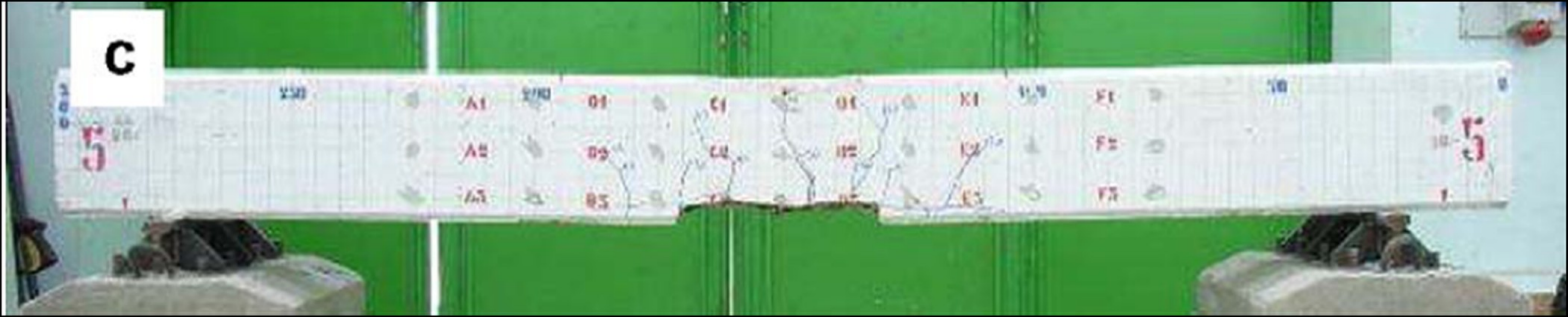
Failure mode of the four-point bending test [34].

Fig 19 shows the mesoscopic damage evolution in the bonding interface at different stiffness reduction stages. It could be seen that at the State I, the mortar below the reinforcement starts to fail in the pure bending portion. At State II, the damaged elements in the pure bending portion have extended to the half of the beam’s height. Above the reinforcement, the length of damaged zone reaches 2/3 of the length of the bending-shear portion; while below the reinforcement, the length of the damaged zone reaches 1/3 of the length of the bending-shear portion. At State III, bending resistance of the beam has been completely lost, which means the fracture of the beam. In the fracture state, the height of the damaged zone reaches 5/7 of the beam’s height in the pure bending portion, and all the elements along the upper interface of reinforcement have failed. Compared with the result of the four-point bending test shown in Fig 20 [34], it can be seen that the failure mode obtained from simulation is consistent with the test result.

Presumably, concave descending of the overall stiffness may be due to the failure in pure bending zone of RC beam, while the plateau descending of the overall stiffness is probably due to continuous extension of the damaged zone along the axial of reinforcement.

Further research is conducted for the influence of mesoscopic damage evolution on the overall effective stiffness.

**Fig 21:**
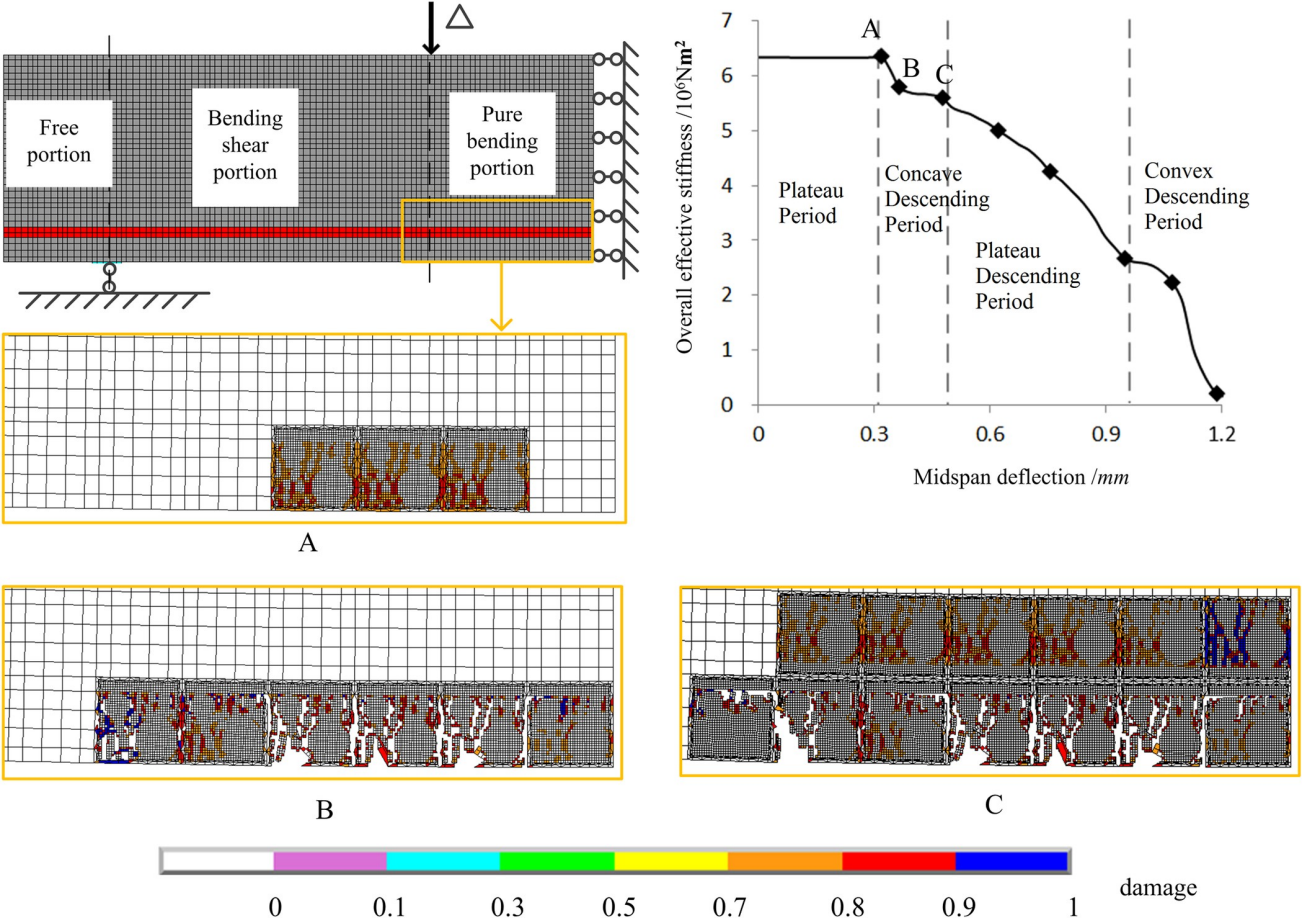
Three damage states in the concave descending portion of the overall effective stiffness. There are three damage states of A, B and C, which correspond to the points A, B and C that are located on concave descending period of the curve of overall effective stiffness.

Fig 21 shows the three damage states, points A, B and C in concave descending stage of the overall effective stiffness. For State A, tensile damage occurs in the pure bending portion. No mortar element is damaged in this state though the damage level is relative high in mortar elements near the coarse aggregates. For State B, tensile damage is distributed largely in the mortar elements below the steel rebar in the pure bending portion and bonding failure occurs initially in this area. For State C, the damaged zone extends to the upper interface of reinforcement and part of the interface below the reinforcement has failed.

**Fig 22:**
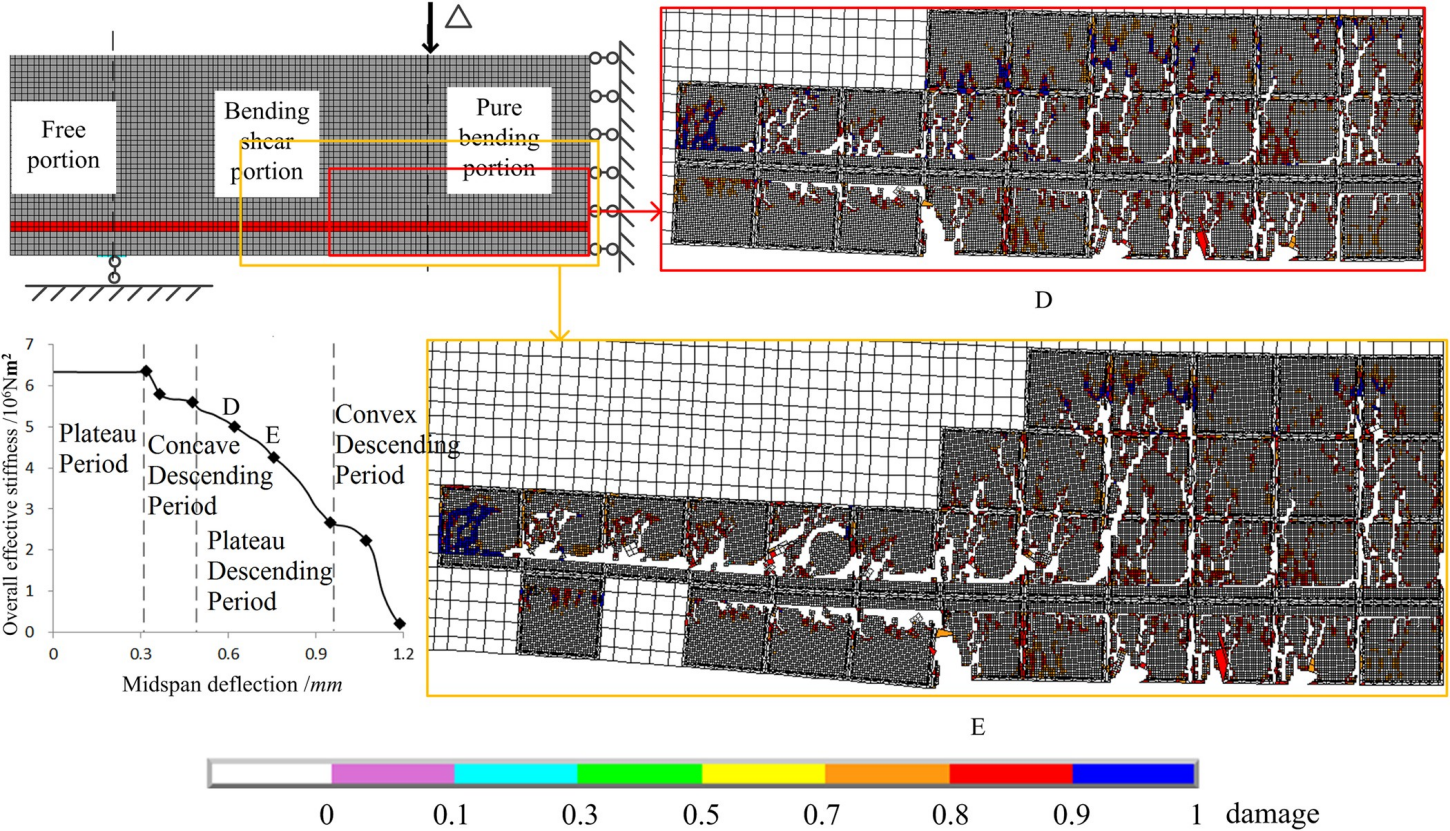
Two damage states in the steady descending portion of the overall effective stiffness. There are two damage states of D and E, which correspond to the points D and E that are located on steady descending period of the curve of overall effective stiffness.

Fig 22 shows the two damage states, points D and E in the steady descending period of the overall effective stiffness curve. For State D, the damaged zone extends to 2/5 of the beam height in the pure bending portion and 1/3 in the bending-shear portion. For State E, the damaged zone extends to the beam’s height of 3/5 in the pure bending portion and 2/3 in the bending-shear portion. It could be inferred that the decline of the overall effective stiffness may be due to the extension of the damaged zone towards the upper compression zone in the pure bending portion, and the extension of the damaged zone along the axial steel rebar in the bending-shear portion.

**Fig 23:**
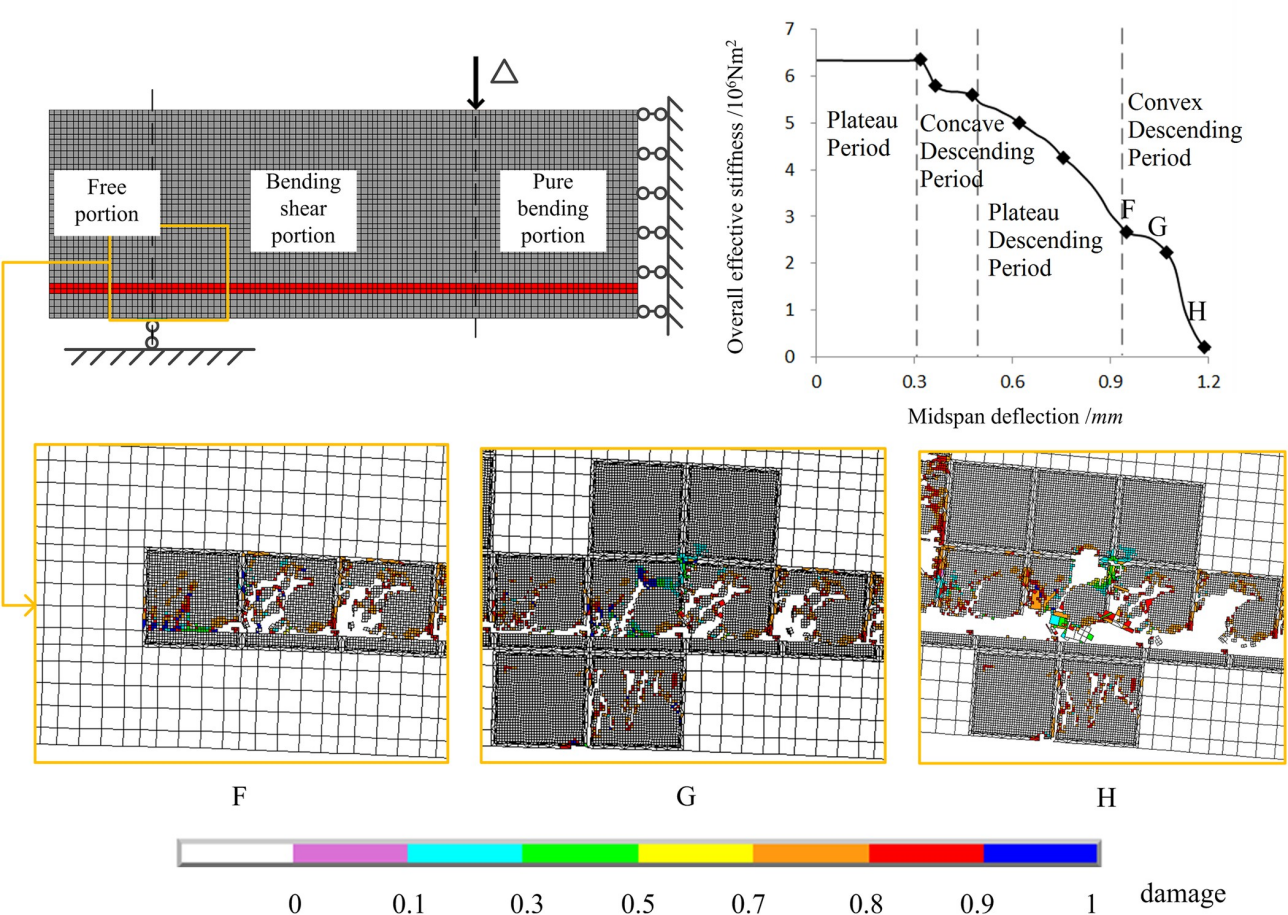
Three damage states in the convex descending portion of the effective stiffness. There are three damage states of F, G and H, which correspond to the points F, G and H that are located on convex descending period of the curve of overall effective stiffness.

Fig 23 shows the three states, points F, G and H in the convex descending period of the overall effective stiffness. For State F, damage is observed in the mortar elements on the upper surface of the reinforcement above the support. For State G, the damaged zone extends to the free end. For State H, the upper surface of the reinforcement fails, and the damaged zones coalescence along the steel rebar. From State F to State G, the evolution of the overall effective stiffness tends to be steady, which is probably because the damage of mortar at the upper surface of the reinforcement blocks the coalescence of the damaged zones in the bonding interface between concrete and steel rebar. The rapid decrease of overall effective stiffness from State G to State H may be due to the failure of mortar elements on the bonding interface. The coalescence of damaged zones leads to fracture of the beam.

## 5 Conclusions

The following conclusions are obtained in this paper:

1. A multi-scale finite element model for RC structure is established based on the adaptive mesh encryption method, in which the interfacial mesoscopic damage evolution is simulated. At the mesoscopic level, the material is considered as a three-phase heterogeneous material consisting of mortar, coarse aggregates and steel rebar. The distribution of coarse aggregates in the mortar was determined by random Monte-Carlo method. The mesoscopic damage is categorized into tensile damage and compressive-shear damage, of which the damage thresholds are defined by the maximum tensile strain criterion and Mohr-Coulomb criterion respectively. Compared with the traditional simulation method, the proposed multi-scale modeling method considering mesoscopic damage evolution is more suitable in the simulation of the deterioration of macroscopic performance of the actual RC structure.
2. The stress-strain curves and failure modes of concrete under uniaxial tension and uniaxial compression are obtained by the proposed simulation method considering the mesoscopic damage in the bonding interface. The proposed algorithm is verified by comparison with the results given by specification and literature.
3. The multi-scale algorithm developed in this paper is proved to be effective in the simulation of the failure process of a four-point bending RC beam, and the influence of mesoscopic damage of bonding interface on the macroscopic performance deterioration of the RC beam is obtained. Macroscopic performance deterioration and the mesoscopic damage evolution could be divided into four stages. In the first stage, the tensile force increases linearly with the deflection of the beam. In the second stage, the damaged zone appears below the reinforcement in the pure bending portion of the beam, and the tensile force increases nonlinearly. In the third stage, the damaged zone in the pure bending portion extends towards the compression zone, and meanwhile the damaged zone above the reinforcement in the bending-shear portion extends along the steel rebar. The overall effective stiffness of the beam reduces by 50%, and the drawing force reaches the peak value when the damaged zone extends to half of the bending-shear portion. In the fourth stage, the compression-shear damage in the upper bonding interface above the support blocks the coalescence of damaged zone along the steel rebar, and meantime drawing force starts to decrease. The results show that when the bonding strength is relatively low, the failure of the bending-shear portion would be caused by coalescence of the damaged zone along the axial direction, which leads to the significant reduction of macroscopic bending performance of the RC structure.

## 6 Discussions

### 6.1 Comparison of results with published literatures

**Fig 24:**
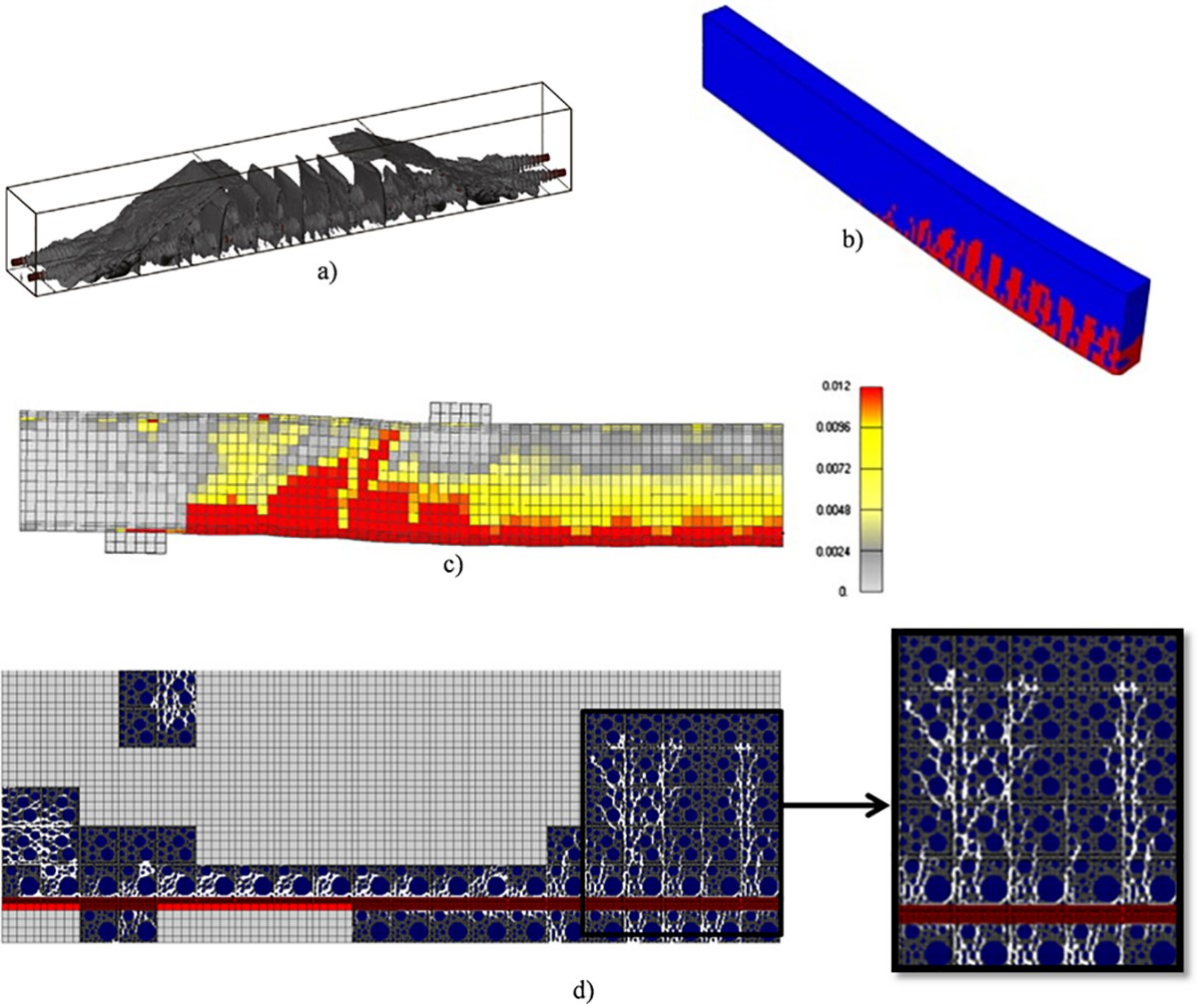
Simulation of debonding or slip of four-point bending RC beam. a) The result given in ref [35]. b) The result given in ref [36]. c) The result given in ref [37]. d) The result in this paper. The first three simulation results didn’t consider the influence of the distribution of coarse aggregates totally; while the last result could present the influence of the distribution of coarse aggregates on crack propagation.

Numerical techniques that replace crack defects with the degradation of material stiffness have been investigated for simulating the fracture behavior of concrete indirectly, owing to the methods’ good compatibility with FEM. One such representative method for concrete material is the damage model, which enables the simulation of 2D or 3D crack propagation behavior including the debonding and slip between concrete and reinforcement. However, concrete is regarded as a homogenous material in most research in this field. For example, in ref [35–37], crack propagation in the bonding interface of four-point bending RC beam is shown in Fig 24A, 24B and 24C. From the Fig 24 we can see, an outstanding feature of the methods mentioned above is the ability to reproduce the geometry and distribution of arbitrary cracks in reinforced concrete. But in fact, the distribution of coarse aggregates also affects damage evolution in the reinforced concrete, which almost wasn’t considered in the published literatures. Therefore, in this paper, the proposed method can present random distribution of coarse aggregates and simulate bond behavior of RC structure considering inhomogeneity of concrete in mesoscopic level, as shown Fig 24D. From the results in section 5 we can see, the existence and distribution of coarse aggregates truly influence the bond behavior of RC structure and the trajectory of crack propagation.

### 6.2 Limitations and future works

The multi-scale numerical simulation method can effectively simulate the mechanical properties of RC structure. This study is only a limited part of it. The following work can be carried out in the future:

1. This paper focuses only on reinforced concrete beams. In order to study the evolution of mesoscopic damage and its influence on the response of the whole structure, it is necessary to further develop the adaptive mesh encryption technology and conduct multi-scale modeling for the complete structure.
2. The model established in this paper is a two-dimensional model. It will be more accurate to study the microscopic damage with three-dimensional solid model. But there are many difficulties, such as: the development of effective three-dimensional aggregate production and placement algorithm because the existed method to judge overlapping of 3D aggregate is inefficient; the development of three-dimensional adaptive algorithms for large scale structures.
3. In fact, the damage accumulation and the extension of damaged zone in the interface zone are closely related to the loading rate and the loading mode. Therefore, it is necessary to study on the meso-damage evolution of the interface zone under the action of earthquake or fatigue load.

## Supporting information

S1 File Data generated by Monte Carlo simulation. The data are size and distribution of coarse aggregates. They are generated by Monte Carlo simulation in Matlab software and then are inputted into ANASYS software to produce the mesoscopic model of RC beam in section 4.
(TXT)

S2 File Data for macro performance deterioration of RC beam. The data are initial data to draw Fig 16, Fig 17 and Fig 18.
(XLSX)

## References

[1] Cao YYY, Yu QL. Effect of inclination angle on hooked end steel fiber pullout behavior in ultra-high performance concrete. Compos Struct. 2018 10;201:151–60. WOS:000440941200015. English.

[2] Abdallah S, Fan MZ, Rees DWA. Bonding Mechanisms and Strength of Steel Fiber-Reinforced Cementitious Composites: Overview. Journal of Materials in Civil Engineering. 2018 3;30(3). WOS:000422885000006.

[3] Visintin P, Oehlers DJ, Sturm AB. Mechanics solutions for deflection and cracking in concrete. Proc Inst Civil Eng-Struct Build. 2016 12;169(12):912–24. WOS:000388197600004. English.

[4] Choi CK, Cheung SH. Tension stiffening model for planar reinforced concrete members. Comput Struct. 1996 4;59(1):179–90. WOS:A1996TU23200016. English.

[5] Wu ZH, Yoshikawa H, Tanabe TA. TENSION STIFFNESS MODEL FOR CRACKED REINFORCED-CONCRETE. J Struct Eng-ASCE. 1991 3;117(3):715–32. WOS:A1991EZ15600006. English.

[6] Poli SD, Diprisco M, Gambarova PG. COVER AND STIRRUP EFFECTS ON THE SHEAR RESPONSE OF DOWEL BAR EMBEDDED IN CONCRETE. ACI Struct J. 1993 Jul-Aug;90(4):441–50. WOS:A1993MK75600011. English.

[7] Deipoli S, Diprisco M, Gambarova PG. SHEAR RESPONSE, DEFORMATIONS, AND SUBGRADE STIFFNESS OF A DOWEL BAR EMBEDDED IN CONCRETE. ACI Struct J. 1992 Nov-Dec;89(6):665–75. WOS:A1992JY21100008. English.

[8] Mohammadi T, Wan BL, Harries KA. Bond-slip behavior of fiber-reinforced polymer/concrete interface in single shear pull-out and beam tests. J Reinf Plast Compos. 2016 3;35(5):375–86. WOS:000372303800002. English.

[9] Akramifard HR, Mirzadeh H, Parsa MH. Estimating interface bonding strength in clad sheets based on tensile test results. Mater Des. 2014 12;64:307–9. WOS:000342681600039. English.

[10] Yang ST, Huang WP, Liu YL. Push-out test to study bond properties of mortar-concrete interface. Mag Concr Res. 2014;66(21):1104–15. WOS:000345853300004. English.

[11] Yoo DY, Park JJ, Kim SW, Yoon YS. Influence of reinforcing bar type on autogenous shrinkage stress and bond behavior of ultra high performance fiber reinforced concrete. Cem Concr Compos. 2014 4;48:150–61. WOS:000334484200017. English.

[12] Murcia-Delso J, Shing PB. Bond-Slip Model for Detailed Finite-Element Analysis of Reinforced Concrete Structures. J Struct Eng. 2015 4;141(4):10. WOS:000351475000009. English.

[13] Mirza SM, Houde J. STUDY OF BOND STRESS-SLIP RELATIONSHIPS IN REINFORCED-CONCRETE. Journal of the American Concrete Institute. 1979;76(1):19–46. WOS:A1979GE67900003. English.

[14] Kankam CK. Relationship of bond stress, steel stress, and slip in reinforced concrete. J Struct Eng-ASCE. 1997 1;123(1):79–85. WOS:A1997VZ03800010. English.

[15] Tassios TP, Koroneos EG. LOCAL BOND-SLIP RELATIONSHIPS BY MEANS OF THE MOIRE METHOD. Journal of the American Concrete Institute. 1984;81(1):27–34. WOS:A1984SB87700004. English.

[16] Bakir PG, Boduroglu HM. Nonlinear analysis of beam-column joints using softened truss model. Mech Res Commun. 2006 Mar-Apr;33(2):134–47. WOS:000233710800002. English.

[17] Wang TJ, Hsu TTC. Nonlinear finite element analysis of concrete structures using new constitutive models. Comput Struct. 2001 12;79(32):2781–91. WOS:000172936200001. English.

[18] Lundgren K, Gylltoft K. A model for the bond between concrete and reinforcement. Mag Concr Res. 2000 2;52(1):53–63. WOS:000086609900007. English.

[19] Salem HM, Maekawa K. Pre- and postyield finite element method simulation of bond of ribbed reinforcing bars. J Struct Eng. 2004 4;130(4):671–80. WOS:000220530800015. English.

[20] Sun B, Wang X, Li ZX. Meso-scale image-based modeling of reinforced concrete and adaptive multi-scale analyses on damage evolution in concrete structures. Comput Mater Sci. 2015 12;110:39–53. WOS:000362010800006. English.

[21] Soh CK, Chiew SP, Dong YX. Damage model for concrete-steel interface. J Eng Mech-ASCE. 1999 Aug;125(8):979–83. WOS:000081610800016. English.

[22] Alfano G, Sacco E. Combining interface damage and friction in a cohesive-zone model. Int J Numer Methods Eng. 2006 10;68(5):542–82. WOS:000241535000003. English.

[23] Mi Y, Crisfield MA, Davies GAO, Hellweg HB. Progressive delamination using interface elements. J Compos Mater. 1998;32(14):1246–72. WOS:000075015800001. English.

[24] Dominguez N, Ibrahimbegovic A. A non-linear thermodynamical model for steel-concrete bonding. Comput Struct. 2012 9;106:29–45. WOS:000307148900004. English.

[25] Nguyen VP, Stroeven M, Sluys LJ. Multiscale failure modeling of concrete: Micromechanical modeling, discontinuous homogenization and parallel computations. Comput Meth Appl Mech Eng. 2012;201:139–56. WOS:000298570500011. English.

[26] Fang CQ, Lundgren K, Chen LG, Zhu CY. Corrosion influence on bond in reinforced concrete. Cem Concr Res. 2004 11;34(11):2159–67. WOS:000224474200026. English.

[27] Zhang NL, Guo XM, Zhu BB, Guo L. A mesoscale model based on Monte-Carlo method for concrete fracture behavior study. Sci China-Technol Sci. 2012 12;55(12):3278–84. WOS:000313766100004. English.

[28] Tang CA, Wan WC. Damage and fracture of concrete- numerical experiments Beijing: Science Press; 2003. Chinese.

[29] Jiang JJ, Lu XZ, Lie LP. Finite element analysis of concrete structures Beijing: Tsinghua University Press; 2005. Chinese.

[30] Sun B, Li ZX. Adaptive image-based method for integrated multi-scale modeling of damage evolution in heterogeneous concrete. Comput Struct. 2015 5;152:66–81. WOS:000352327000006. English.

[31] Peng B, Zheng W. Study on the test method of concrete strength in uniaxial direct tension. Journal of Hunan University(Natural Sciences). 2004;31(2):79–83. Chinese.

[32] Housing and urban-rural development of the People's Republic of China. Code for design of concrete structures (GB50010-2010). China. 2010. Chinese.

[33] Gao R. The numerical simulation of mesoscopic damage and failure process of concrete. Dalian: Dalian University of Technology; 2008. Chinese.

[34] Rinaldi Z, Imperatore S, Valente C. Experimental evaluation of the flexural behavior of corroded P/C beams. Constr Build Mater. 2010 11;24(11):2267–78. WOS:000280778700029. English.

[35] Kurumatani M, Soma Y, Terada K. Simulations of cohesive fracture behavior of reinforced concrete by a fracture-mechanics-based damage model. Eng Fract Mech. 2019 2;206:392–407. WOS:000454511700024. English.

[36] Wang XM, Zhou CW. Numerical investigation for the flexural strengthening of reinforced concrete beams with external prestressed HFRP sheets. Constr Build Mater. 2018 11;189:804–15. WOS:000449133200077. English.

[37] Ozbolt J, Bosnjak J, Periskic G, Sharma A. 3D numerical analysis of reinforced concrete beams exposed to elevated temperature. Eng Struct. 2014 1;58:166–74. WOS:000331433000018. English.

